# Exploring targeted preventive health check interventions – a realist synthesis

**DOI:** 10.1186/s12889-023-16861-8

**Published:** 2023-10-05

**Authors:** Nanna Bjørnbak Christoffersen, Freja Ekstrøm Nilou, Trine Thilsing, Lars Bruun Larsen, Jane Nautrup Østergaard, Marie Broholm-Jørgensen

**Affiliations:** 1 Research Program On Health and Social Conditions, National Institute of Public Health, University of Southern Denmark, Copenhagen, Denmark; 2 Research Unit of General Practice, University of Southern, Odense, Denmark; 3 Steno Diabetes Center Zealand, Holbæk, Denmark; 4 Steno Diabetes Center Aarhus, Aarhus University Hospital, Aarhus, Denmark

**Keywords:** Realist synthesis, Preventive health checks, Intervention research, Denmark, Participatory research, Chronic disease

## Abstract

**Background:**

Preventive health checks are assumed to reduce the risk of the development of cardio-metabolic disease in the long term. Although no solid evidence of effect is shown on health checks targeting the general population, studies suggest positive effects if health checks target people or groups identified at risk of disease. The aim of this study is to explore why and how targeted preventive health checks work, for whom they work, and under which circumstances they can be expected to work.

**Methods:**

The study is designed as a realist synthesis that consists of four phases, each including collection and analysis of empirical data: 1) Literature search of systematic reviews and meta-analysis, 2) Interviews with key-stakeholders, 3) Literature search of qualitative studies and grey literature, and 4) Workshops with key stakeholders and end-users. Through the iterative analysis we identified the interrelationship between contexts, mechanisms, and outcomes to develop a program theory encompassing hypotheses about targeted preventive health checks.

**Results:**

Based on an iterative analysis of the data material, we developed a final program theory consisting of seven themes; *Target group; Recruitment and participation; The encounter between professional and participants; Follow-up activities; Implementation and operation;* *Shared understanding of the intervention;* and *Unintended side effects.* Overall, the data material showed that targeted preventive health checks need to be accessible, recognizable, and relevant for the participants’ everyday lives as well as meaningful to the professionals involved.

The results showed that identifying a target group, that both benefit from attending and have the resources to participate pose a challenge for targeted preventive health check interventions. This challenge illustrates the importance of designing the recruitment and intervention activities according to the target groups particular life situation.

**Conclusion:**

The results indicate that a one-size-fits-all model of targeted preventive health checks should be abandoned, and that intervention activities and implementation depend on for whom and under which circumstances the intervention is initiated. Based on the results we suggest that future initiatives conduct thorough needs assessment as the basis for decisions about where and how the preventive health checks are implemented.

**Supplementary Information:**

The online version contains supplementary material available at 10.1186/s12889-023-16861-8.

## Introduction

Preventive health checks are assumed to reduce the risk of developing cardio-metabolic diseases and, other non-communicable diseases, respectively or to increase the chance of diagnosing a high risk of disease earlier [1–5]. Such health checks might compromise physical examination, survey and follow-up of people who are considered of high risk or disease. Although, individual-oriented preventive health checks targeting the general population have not shown solid evidence [6–8], a number of studies suggest positive effects if the health checks target people or groups identified as being at high risk of disease [9–15]. Several models for preventive health checks targeting people at increased risk of cardio-metabolic disease have been developed and evaluated within the last decade [16, 17]. For example, the Dutch INTEGRATE study, which explored the effectiveness and cost-effectiveness of the combination of an online risk estimation and a tailored lifestyle intervention in general practice, proved both feasible and effective [16]. Despite great variations in the structure, content, target group and implementation of preventive health checks, studies indicate that a systematic detection of people or groups at increased risk may be relevant [1–3].

In the present study we differentiate between *general preventive health checks*, where health checks are offered to the general population (in a certain age group regardless of risk) and *targeted preventive health checks*, in which only individuals or a group of people at high risk are offered the health checks. People eligible for targeted preventive health checks may be identified either from indicated or selective prevention [18]. Indicated prevention is when risk is identified by individual risk assessment of total risk (risk algorithms) or single risk factors (biomarkers, etc.). Selective prevention is based on existing evidence about the specific risk of groups of people (men, social housing areas, low socioeconomic status, physical and psychological disabilities etc.). Targeted preventive health checks normally consist of three central components: 1) a risk assessment that identifies the target population, 2) a health check followed up by a health conversation, and 3) follow-up activities [19]. The health check typically consists of several clinical exams, such as measurements of weight, height, lung function and blood pressure as well as various blood tests. The results provide insights into the patient’s general state of health and potential risk and unrecognised illness may be detected [19]. The health check is usually followed by a health conversation, where the patient is informed about the results and given advise about possible pharmacological treatment or relevant changes in health behaviour [20]. Generally, the preventive intervention also includes one or more preventive or curative follow-up activities. Follow-up activities can be pharmacological treatment, a second health check following up on the results from the first health heck, civil society-based activities, such as activities in local sports clubs, or patient or self-management education, such as courses about diet, physical activity, smoking habits, stress, or sleep or or a combination of these [6]. Because studies indicate that the targeted preventive health checks may have positive health effects [1–3, 21], we focus on this type of intervention in this study. Henceforward, we will use the term preventive health checks.

Due to the complexity of implementing health checks they are rarely implemented according to protocol [22, 23]. The most frequently reported and important barriers for implementation of preventive health checks are lack of time, staffing, funding, and training needs for those delivering the health checks [24, 25]. However, only few studies have examined the implementation of health checks. Hence, important information on how, why, for whom and under what circumstances preventive health checks may work lies as tacit knowledge among health professionals, project managers and participating patients etc. This knowledge, thus not available publicly, is very important for the overall understanding of whether and how preventive health checks work. Furthermore, it is imperative for the future design and implementation of preventive health checks that the compound knowledge about these interventions is described and brought into play.

Therefore, the aim of this study is to identify important elements relevant for the planning and implementation of possible future preventive health checks. To this end we explore why and how preventive health checks work towards adverse health behaviour in high-risk groups, for whom they work, and under which circumstances they can be expected to work.

This study conveys knowledge and findings from a Danish report [26].

## Methods and materials

### Design

This study is designed as a realist synthesis, because this design allows us to gather knowledge from a wide range of preventive health check interventions, which have been carried out over the recent years across different countries, as long as either the mechanisms or the contexts are relevant to the aim of the synthesis. The design further entails the development of a program theory encompassing hypotheses about the working mechanisms of preventive health checks. The present study synthesises evidence from three literature studies (quantitative literature, qualitative literature and grey literature respectively), 17 interviews and four workshops.

The realist synthesis originates from a critique of quantitative meta-analyses and systematic reviews [27]. Besides including explorations of context, the realist synthesis differs from the more traditional systematic review by being theory-based and aiming to develop a program theory. A program theory is defined as explicit assumption (theories) about how an intervention contributes to a chain of intermediate results and finally to the intended or observed outcomes [28]. Within the realist synthesis methodological approach program theories consist of a number of CMO-configurations, which cover the interrelationship between context, mechanisms and outcomes [29]. The CMO-configurations describe a generative approach to causality, where specific contextual factors trigger (or do not trigger) mechanisms. The causal potential is then realized in form of a causal outcome [30]. Mechanisms are partly the resources the intervention activities offer, such as a preventive health check, and partly the response the activities trigger in the participants. In this way, the activities “work” through participants’ reasoning, which they act upon [31, 32]. This understanding posits that individuals change their behavior because of the resources made available to them, such as an invitation to a health check or follow-up activities, which prompt them to reflect, reason and act differently. Contexts are the circumstances that activate mechanisms and are defined as the pre-existing social, cultural, and physical conditions which allow the mechanism to come into operation [32]. Outcomes are the intended and unintended consequences of the intervention activities. Importantly, intervention activities do not produce outcomes in themselves according to the realist methodological approach, but the activities offer opportunities which may – or may not- trigger action by means of the participants’ reasoning and capacity to act [32]. This means, that outcomes of preventive health checks may differ depending on the target group and the contextual conditions in which the health check is implemented.

**Fig. 1:**
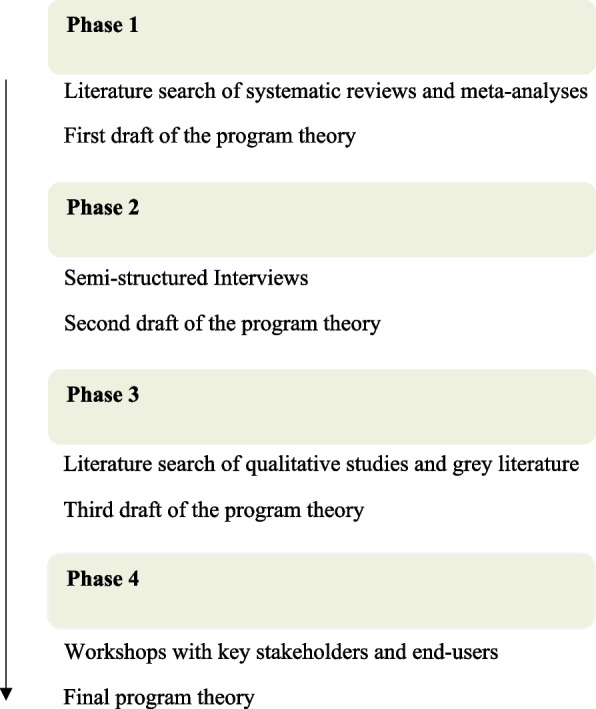
Study design

In this realist synthesis we used an iterative stakeholder-driven approach with four phases, each including collection and analysis of empirical data: 1) Literature search of systematic reviews and meta-analysis, 2) Interviews with key-stakeholders, 3) Literature search of qualitative studies and grey literature, and 4) Workshops with key stakeholders and end-users (see Fig. 1 for an overview of the four phases). The order of the phases permitted an iterative move back and forth between the literature and the empirical material, which allowed us to question and deepen the findings from the literature in the interviews with stakeholders. Based on the results of the first phase an initial draft of the program theory was developed. The following three phases were used to adjust, elaborate or confirm different elements of the program theory. In the following section, the four phases will be elaborated.

#### Phase 1: Literature review of systematic reviews and meta-analyses

In the first phase of the study, we carried out a literature review focusing on quantitative systematic reviews and meta-analyses, with the aim of gaining insight into the international literature in the field of research. To this end, we searched the databases Embase, PsychINFO, Cochrane Central Database and Global Health using the following MESH terms: Health Check, Chronic Disease, Primary Care, High risk, Targeted (see supplementary file 1). These terms were combined with methodological search terms for systematic reviews and meta-analyses. Studies were included if: 1) the type of publication was a systematic review or a meta-analysis, 2) the publication was written in English; 3) the publication date was after 1990, and 4) the study participants were adults. The search was conducted in May 2021.

#### Phase 2: Semi-structured Interviews

In phase 2, we conducted semi-structured qualitative interviews with professionals with experience within the field of preventive health check interventions in Denmark. The purpose of these interviews was to activate the knowledge and experience of the interviewees which have not necessarily been published in scientific articles or reports [33, 34].

Recruitment of interviewees was based on an overview of all Danish preventive interventions, that included health checks within the last 40 years—both targeted preventive interventions and interventions offered to the general population. The interviewees were then selected based on maximum variation with regard to the different interventions, and the role they had in the intervention [35]. Thus, managers, researchers, project staff and different health professionals were all among the interviewees. We invited 23 people to participate in interviews by e-mail and ended up conducting 16 interviews with a total of 17 interviewees, since one interview was carried out as a group interview. The interviews were carried out in September and October 2021. See Table 1 for an overview of interviewees’ roles in the different preventive interventions.

**Table 1:** Overview of interviewees

Role	**Municipal/regional manager**	**Researcher**	**Project worker**	**General Practitioner (GP)**	**Municipal Health professional**
Number of interviewees	2	6	2	4	3

The interviews lasted approximately one hour. Some interviews took place face-to-face, and others were carried out online via zoom or as telephone interviews. The interview guide was developed based on the first draft of the program theory and included the following topics: Intervention elements; Choice of target group; Recruitment; Retention of participants; Implementation; Evaluation; Mental health. The semi-structured design of the interviews left room for the interviewee’s own conceptualization of preventive health checks [36]. All interviews were digitally recorded and transcribed verbatim.

#### Phase 3: Literature review of grey literature and qualitative studies

In phase 3, we carried out two literature reviews and two separate analyses, with the overall aim of further adjusting and qualifying the program theory. Furthermore, the purpose of this phase was to sound the depths of some of the themes that emerged in the interviews, such as providing in-depth information about selected target groups and unintended side effects (see the supplementary file 2). Therefore, the search words from phase 1 were adjusted to accommodate the findings from phase 2.

The search for grey literature of Danish preventive health check interventions (literature that is not formally published in books or peer reviewed journal articles, including project reports and evaluations [37]) was identified through websites and open searches on Google. Furthermore, we collected grey literature through phase 2, for example when interviewees provided us with evaluation reports from the different interventions [38].

In phase 3, we additionally conducted a literature review of peer-reviewed qualitative studies to include insights into the target group’s experiences of participation. The search for qualitative literature was focused on targeted preventive health check interventions. The systematic search for literature was carried out in the databases Embase, PsychINFO, Cochrane Central Database and Global Health (see supplementary file 1) in November–December 2021. Studies were included if 1) they were conducted in Scandinavia, the Netherlands, and/or Great Britain, with the intent to include studies based on a study population and primary health care systems that resemble a Danish context [39], 2) the type of publication was a qualitative study, 3) the publication date was after 1990, and 4) if the study participants were adults.

#### Phase 4: Workshops with key stakeholders and potential end-users

In phase 4 we conducted workshops with key stakeholders, such as researchers, health professionals and potential end-users, with the aim of qualifying and refining the pre-final program theory. Conducting workshops enabled us to obtain stakeholders’ responses to the program theory and on this basis refine the program theory to qualify it and make it relevant and meaningful for those who deliver and receive the intervention [40]. We conducted a total of four workshops from February to April 2022, of which the first three were held with professionals and the last workshop with potential end-users (See Table 2: Participants in workshops).

**Table 2:** Participants in workshops

Role	**Municipal/regional manager**	**Researcher**	**Project worker**	**General Practitioner (GP)**	**Municipal Health professional**	**End-user**
Number of workshop participants	1	5	3	1	3	3

Workshops with professionals lasted 2,5 h and had four to five participants each. We recruited participants based on the overview of Danish health check interventions (see phase 2) including some of the interviewees from phase 2. We selected the participants based on maximum variation regarding the different interventions and their role in the intervention [35]. The workshops were structured into two activities. In the first activity the participants presented and discussed their immediate assessment of the program theory based on the questions 1) what is consistent with your knowledge and experience, 2) what is inconsistent with your knowledge and experience, and 3) what have we overlooked? In the second activity, the participants were asked to reflect on the barriers for future targeted preventive health checks followed by a group discussion about potential solutions to these barriers. We audio-recorded the workshops and took comprehensive notes.

The workshop with end-users aimed to explore potential end-users’ understanding of health and health checks. Initially we attempted to recruit potential end-users through a range of local community groups on Facebook, however this was not successful. Instead, we ended up recruiting potential end-users through Steno Diabetes Centre Zealand’s user panel. The participants in this workshop were women aged 50–65 years. The workshop lasted two hours.

Similar to the workshops with professionals, this workshop was structured around two activities. The first activity was a group interview aiming to get insights into the participants’ understandings of health and experiences with health checks. The second activity was based on a discussion about a vignette describing a fictitious person who was invited to a preventive health check [41, 42].

### Data analysis

Adhering to key analytical principles within realist evaluation, an iterative approach to data analysis was adopted across each phase [43]. Using multiple data sources as well as insights from the project group (NBC, FN, TT and MBJ), the analysis of data moved iteratively between the drafts of program theories and the data to identify the mechanisms operating within a context that led to an outcome [44]. The analytical approach additionally gained inspiration from the principles of collaborative analysis which meant that analytical themes were discussed in the project group in order to build a collective knowledge and understanding of the shared body of data [45].

Identification of the initial program theory was based on the findings from phase 1. We carried out a thematic analysis of the identified systematic reviews and meta-analyses [46]. *If–then* sentences were applied as an analytical tool to identify contexts, mechanisms and outcomes based on the results of the preventive health checks in the included literature. Thus, to construct conditions for possible effects (outcomes) of the included literature, each member of the project group developed series of *if–then* sentences [47]. The constructed *if–then* sentences were discussed in the project group and thematized. Based on this analysis process we developed the first draft of the program theory (see the supplementary file 2).

In phase 2 and 3 we tested and continuously refined the program theory in the following procedure. In phase 2 we carried out a thematic analysis of the interviews [46]. The analytical process was similar to the abductive analysis approach which rendered the possibility to remain open to surprising element in or aspects of the empirical object and at the same time move between the empirical material and the theory [48]. Themes were subsequently discussed and reviewed in the group. The analysis of the interview data brought forth the following themes: *Target group, Recruitment, Methods, Organizational framework, Consistency in the intervention, Recognizability, Availability and relevance, The encounter with the participants.* Based on a realist approach to analysis, we compared the new themes with the first draft of the program theory from phase 1 and considered how the themes gave rise to changes, nuances and supplements to the CMO-configurations [43]. Based on this, we developed the second draft of the program theory (see the supplementary file 2).

The studies from phase 3 were thematically coded using the program NVivo. We created initial codes based on the themes from the second draft of the program theory, while new codes were added during the analysis. After coding the studies, we considered whether the analysis gave rise to adjustments or supplements to the program theory or confirmed or contradicted hypotheses in the second draft of the program theory [43]. Based on the analysis of the literature from phase 3 we developed a third draft of the program theory (see the supplementary file 2).

The final draft of the program theory involved integrated analysis and interpretation by iterative testing and refining the CMO statements during the workshops in phase 4.

### Ethical considerations

Written and oral consent was obtained from each study participant prior to interviews and workshops and all participants were informed of the ethical principles involved regarding confidentiality and anonymity. However, because the study is based on well-known Danish preventive health check interventions, it is not possible to completely anonymize the professional study participants. This means that the professional study participants may be able to recognize themselves or others in the quotes due to their role in an intervention. All professional study participants have been made aware of this proviso before they participated in the study.

## Results

**Fig. 2:**
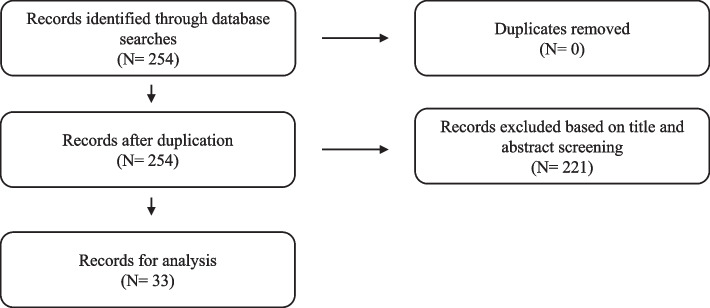
Screening process of phase 1

In phase 1 the total literature search resulted in 254 publications. After abstract screening 33 publications were included in the final analysis (See Fig. 2 for the screening process of phase 1).

The search included 26 systematic reviews and seven meta-analyses. Analysis of the data gathered during phase 1 resulted in the development of six theoretical propositions making up the initial program theory (see the supplementary file 2).

**Fig. 3:**
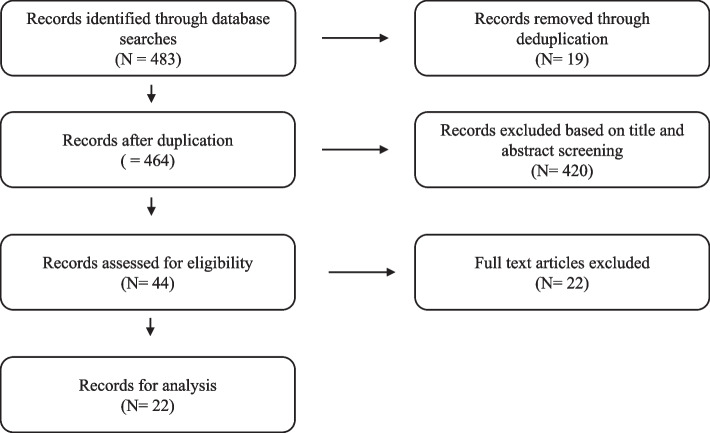
Screening process of phase 3

The literature search of grey literature in phase 3 yielded 10 reports. These reports included effect- and process evaluations of Danish preventive initiatives. The literature search for qualitative studies in this phase yielded 22 relevant studies (see Fig. 3: The screening process of phase 3).

A detailed table of alle included studies is available in supplementary file 4.

The final program theory consists of seven themes; 1) *Target group; 2) Recruitment and participation; 3) The encounter between professional and participants; 4) Follow-up activities; 5) Implementation and operation; 6) Shared understanding of the intervention;* 7) *Unintended side effects.* All themes apart from *Target group* and *Unintended side effects* contain CMO-configurations that illustrate the basic assumption of how and under which circumstances targeted preventive health checks are expected to work (see Table 3).

**Table 3:** Matrix of context-mechanism-outcome configurations that make up the program theory

Theme	Context	Mechanism	Outcome	Supporting research/evidence (reference nr)
**Recruitment and participation**	➣ Illness among relatives or friends ➣ Awareness of own health and risks ➣ Social support	➣ Relevance	➣ Increase in participation	40, 69, 70, 71, 72, 73, 74, 75, 76, 91
	➣ Short geographical distance/proper public transport ➣ Targeted language ➣ Simple booking procedures ➣ Activities accommodated to the needs of the target group	➣ Accessibility	➣ Increase in participation	62, 66, 67, 69, 71, 76, 77, 78, 79, 80, 81, 82, 83
	➣ Recruitment in the local communities ➣ Personal contact in the recruitment process	➣ Recognizability	➣ Increase in participation	69, 71, 72, 77, 83, 84, 85, 86, 87,
**The encounter between the health professionals and the participants**	➣ Professionals who are competent to guide and communicate preventive information ➣ Sufficient time	➣ Focus on participants’ needs and wishes	➣ Initiation of relevant follow-up activities	40, 67, 68, 71, 75, 78, 88, 89, 90
**Follow-up activities**	➣ Short geographical distance/proper public transport ➣ Implementation in local context ➣ Activities accommodated to the needs of the target group	➣ Relevance ➣ Accessibility	➣ Increase in participation	66, 69, 71, 76, 77, 78, 82, 83, 87
**Implementation and operation**	➣ Political and managerial support ➣ Implementation in existing organizations ➣ Sufficient knowledge and cooperation between professionals	➣ Motivation ➣ Relevance	➣ Strengthened implementation and sustainability	67, 68, 69, 82, 87, 91, 92, 94,
**Shared understanding**	➣ Clear definition of aim, design, and outcomes	➣ Shared understanding among professionals of intervention success	➣ Strengthened implementation and evaluation	10, 68, 92, 93, 94,

We present and elaborate the seven themes and the included CMO-configurations in the following sections.

### Theme 1: Target group

Aiming to explore for whom preventive health checks work, we identified a challenge, as those who may benefit most from preventive health cheeks simultaneously are less prone to respond to the invitation, to accept an invitation, and to eventually attend the health check. Due to this challenge, we have not established a CMO-configuration for this theme. In the following paragraphs we will demonstrate the challenge based on the data material.

Target groups at high risk of non-communicable diseases, are also considered to hold the greatest potential for reducing the risk through changes in health behavior [2, 49–51]. Potential target groups are men [52–55], people with ethnic minority backgrounds [56], people with low socio-economic status, for example short education and/or unemployment [57–62], people with estimated high risk [63–66], and people with mental and physical impairment [67–72]. However, the data material reveals that many potential high-risk groups such as residents in local areas of low socio-economic status, people with mental health problems, people with short education and/or unemployment [73–77] are less prone to accept the offer of a preventive health check and eventually show up for the scheduled appointment. Many of these target groups have less flexibility in their everyday lives and challenges and worries which are prioritized over their health [25, 73–79] which may explain the lack of participation.

According to the qualitative data material, it is crucial that future interventions explore and identify the target group’s specific needs. A researcher commented at a workshop:

*We need to start asking the target group about whether they are interested in us helping them – and if so, how. But as it is now, we as researchers go ahead with an intervention with political support. It does not start with the target group expressing a request.*

(Researcher)

Along these lines, it is important to select a clear and defined target group in order to accommodate the intervention to this particular group. Therefore, as part of the preventive initiative a thorough needs assessment should be carried out prior to intervention development and implementation [78].

### Theme 2: Recruitment and participation

This theme contains three CMO-configurations that influence recruitment and participation. The CMO-configurations concerns the mechanisms; Relevance, Accessibility and Recognizability.

#### Relevance

The data material indicates that if the target group experience the preventive health check as relevant in relation to their specific life situation, they are more motivated to participate [51, 80–84]. In one study, not experiencing relevance was highlighted as the most frequent reason for not participating [81]. Lack of relevancy may be due to inadequate information about the content of the preventive health check and what gain the participants may obtain from taking part. Furthermore, if the target group perceive themselves to be healthy, they may not see the point in changing their health behavior and thus they do not consider the health check to be relevant for them [81].

One contextual factor that influences the experience of relevance is the participants’ experience with illness among relatives or friends [51, 82–84]. A participant from the end-user workshop described that her mother had type-2-diabetes (T2D), and thus she had felt a need to be attentive to symptoms and have her health checked regularly. Similarly, a general practitioner (GP) described the impact of experiencing illness in the family:

*When the neighbor has a blood clot in the heart or something similar, it provokes thoughts and motivates, in a way that suddenly, you would choose to change things.* (GP)

Thus, experiences of illness among friends or relatives affect people’s awareness of their own health and risks, which may in turn increase their perception of the relevance in accepting a preventive health check. Additionally, participants’ own experiences with illness and symptoms of illness influence whether they consider preventive health checks to be relevant [51, 85, 86].

Another contextual factor is participants’ social network. Several studies emphasize the importance of involving the local and social network as part of the recruitment strategy [81, 82]. Thereby, it is possible to inform entire families and groups at the same time, and the participants may inspire each other to take part [81, 82]. Similarly, interviewees and participants in the workshop emphasize the possibility of recruiting men, if they are invited together with their spouses. A health professional participating in an interview explains the benefit of inviting couples or family members together:

*Several times we invited couples, because then they could attend at the same time. So, if you lived together, you got the invitation at the same time and that resulted in some really beneficial conversations For example, when they fill out the questionnaire together and such, both the husband and wife could comment on each other like:’ no, that is not true, it’s more than you have stated’ or ‘it’s a little less’.*

(Health professional)

In the evaluation of the Danish preventive health check intervention *Check Your Health* (Tjek dit Helbred – red), the authors find a higher participation rate among those whose partner also participated in the intervention [80]. Correspondingly, lack of social support is described as a reason for non-participation among groups of people with low socio-economic status [79].

#### Accessibility

The data material shows that if participants experience a preventive health check as easily accessible, then they will be more likely to participate. Overall, the studies in this synthesis indicate that short geographical distance, good means of public transportation and an opportunity for online meetings influence participants experience of accessibility and increases participation [77, 79, 81, 86–88]. However, the interviews indicate that the perception of tolerable geographical distance varies and depends on how far they would normally have to transport themselves, for example when attending their family doctor.

Furthermore, the studies in this synthesis indicate that attention to the target group's health literacy as well as language and visual literacy may increase participation. Among other things we find that adjusting the language to the target group in both the invitation and during the intervention activities may increase the participants’ perception of accessibility and thus increase participation [78, 79, 87, 89]. Adjusting the language involves taking the native language of the target group into account (for example by ensuring the possibility of interpreter services), as well as adjusting specific linguistic phrasings and choice of words to the target group [78, 79, 87, 89]. A recent Danish study found that by adjusting the wording of the invitation to the target group they increased participation by 10 percentage points (from 39,4% to 48,3%) [90].

Another contextual factor that increases accessibility is easy, simple, and straightforward procedures for booking appointments for health checks. Interviewees state that several Danish preventive health check interventions have had long, technical, and cumbersome questionnaires and consent forms, because they have been carried out as research projects, which challenge accessibility and constitute a barrier to participation. An interviewee describes the booking procedure of the intervention he/she evaluated:

*The fact that it was easy to book the health check by clicking on a link or a SMS-code, and when you are in the system, then […] you could postpone your appointment and go directly to the questionnaire. I think that meant a lot [for participation].*

(Evaluator)

Likewise, results from another Danish preventive intervention showed that providing a phone call from the participants’ GP with the purpose of scheduling an appointment, made it more likely for men, people with short educational level and people who had not visited their general practitioner within the last two years to take part in the intervention [91].

Additionally, accessibility concerns adjusting the intervention activities to the needs and preferences of the target group. Both interviewees and workshop participants emphasize the possibility of scheduling the health check outside of normal working hours, allowing participants to attend without taking time off work. These findings are supported by the literature [86, 87, 92, 93]. Several interviewees express a need for preventive health checks to be accommodated to the individual participant’s opportunities and needs. An interviewee describes it as follows:

*Instead of just handing a participant a note about a smoking cessation course, we need to figure out how to help the person to stop smoking. For example, the municipality could be more proactive, make eye contact, follow up, go to the participants’ home, try to figure out why this person has difficulties with quitting smoking. In other words, a more hand-held way.*

(Researcher)

#### Recognizability

According to the data material, implementing the preventive health check in the local context, which is recognizable to the target population, creates trust and thus increases participation [82]. According to several of the interviewees, recruitment of participants may take place at, for instance, pharmacies, libraries, municipal health centers, general practice, or job centers. A Dutch study additionally recommends the use of social housing organizations and NGOs in relation to recruitment of people with low socio-economic status [79]. Although we find concordance regarding implementation in the local context, it varies across the data material of what parts of the local context participants trust.

There may be a relative inequality in participation in favor of those who have a good relationship with or regularly attend the place where the health check is implemented. However, recognizability is not always linked to trust and comfort. During a workshop, an end-user said:

*It is good to get the health check from someone else than your GP, who knows you well anyway. Medical students for example, ask different questions, so it can be good to have someone else’s eyes on you, than those you are used to. But you also have to trust the person. And you do not get along well with everybody.*

(End-user)

Although recognizability often determines whether participants choose to participate or not, some participants are motivated by having the health check done by new or alternative providers. However, it is always important that the participants trust the providers of the health check.

Another contextual factor which is found to increase recognizability is personal contact during the recruitment process. Personal contact can be personalized written invitations, for instance by adding a picture or name to the invitation material, as well as phone-calls and face-to-face inquiries. Studies find that the opportunity to ask questions before accepting to participate, for example via a phone call, increases participation [93–96].

An evaluation of 12 Danish municipality health check interventions targeting socioeconomically disadvantaged groups, showed that phone calls and home visits to the participants are beneficial for recruitment, especially if the recruitment is carried out by fellow citizens or other local peers, who have participated in a similar activity [97]. Other studies also point to the advantages of having people from the local area to recruit socioeconomically disadvantaged participants [79] or participants with ethnic minority background [82]. In this line of thought, a workshop participant said:

*It is important to have ambassadors from the local community. Someone who can ‘translate’ the intervention to the target group. Someone who can make it seem meaningful.*

(Municipal health professional)

### Theme 3: The encounter between professional and participant

This theme concerns the encounter between the professionals who deliver the preventive activities and the participants who receive them. The encounter thus includes both the health check and the follow-up conversation. How the health checks and follow-up conversations are carried out varies between different interventions. In some interventions the professional may be a municipal dietician, physiotherapist or alike, and in others a nurse or a GP.

According to a systematic review examining health professionals’ experiences with preventive initiatives in primary care, the professional’s competencies on how to guide and communicate preventive information to patients are emphasized, and a main barrier is lack of training [25]. A Dutch study exploring patients’ attitudes towards preventive health checks, points out the importance of matching expectations, both before, during and after the encounter [88]. The study shows that participants demand information about the health check, so they know what it entails before attending, that they receive thorough follow-up after getting the results and know where they can address questions afterwards [88].

The data material additionally emphasized that participants’ needs, and wishes are included and taken seriously in the encounter with the professionals. Motivational Interviewing and similar interview techniques were mentioned in interviews and workshops as well as in the qualitative studies [51, 98–100]. Furthermore, all participants in interviews and workshops, researchers, health professionals as well as end-users, strongly suggest including mental health elements in the encounters, focusing on, for example, stress, sleep problems, anxiety, depression, and the like. Encompassing mental health in the encounters will support that relevant follow-up activities are initiated.

Time is another contextual factor influencing participants’ experiences of the encounter. According to the end-users, the duration allotted to the encounter needs to be sufficient for the participant to express his or her needs and worries. A GP who had performed preventive health checks as part of an intervention describes in an interview how the extra time with the patients provided an opportunity to discuss topics, they did not normally have the time to discuss:

*Often there are many things we do not have the time to discuss, for example, how many children does the patient have or their living situation and such. There are people who move up and down the social hierarchy over the years, and they may have functioned well in the past, and then things have gone bad in some way. It is nice to know about these things.*

(GP)

Likewise, a qualitative study exploring preventive health checks targeting people with low socio-economic status supports that the duration of the encounters should be long enough for the professionals to thoroughly explain the test results and the participants to ask in-depth questions [71].

### Theme 4: Follow-up activities

This theme concerns the resources and activities that are launched based on the results of the health check and follow-up conversation. A prerequisite for this theme is that the professional and participant have identified one or more preventive activities relevant for the participant’s everyday life.

Similar to *Recruitment and Participation,* the participation in follow-up activities is enhanced when the activities are easily accessible for the participants for instance by taking place in the participants’ local environment [97]. A systematic review of patient-reported factors associated with uptake and completion of cardiovascular lifestyle behavior change demonstrates that geographical distance and limited options for public transportation constitute a barrier for participation in follow-up activities [77, 79, 81, 86–88]. However, as we demonstrated earlier, participants have different perceptions of whether a certain geographic distance composes a challenge or not.

The time of day the activities are provided may also constitute a barrier for participation according to the interviewees and workshop participants. One end-user suggests that follow-up activities are offered outside normal working hours or at weekends, which is supported by the literature [86, 87, 92, 93].

Some workshop participants suggest close collaboration with local sport clubs or other organizations, to ensure that follow-up activities are long-term rather than time limited.

### Theme 5: Implementation and operation

This theme concerns the terms and conditions for implementation and the operation of the preventive health check. In this theme we focus on the professionals who deliver and operate the intervention.

Political and managerial support is central to the professionals prioritizing time to the activities of the intervention. Prioritization entails allocating the necessary resources such as time, tools, staff, facilities, education etc. In several studies, lack of resources is found to be a major challenge for implementation and operation [24, 25, 79, 92, 101].

Furthermore, political prioritization of prevention influences whether both professionals and participants consider a health check intervention to be relevant. This is emphasized by an interviewee:

*There was a lot of positioning in this [the intervention], for example, positions on whether the health check should be provided by general practice or not. […] So, the whole political context framing this intervention has meant a lot. I think it has affected the GPs’ attitudes towards the intervention. So, some people have just been opposed to it [the intervention]*

(Manager in municipality/region)

The professionals’ experiences of relevance and motivation towards delivering the activities in a preventive health check intervention thus depend on political and managerial focus and prioritization. Interviewees additionally suggest implementing the intervention activities, health check, follow-up conversation and -activities, in relation to organizations that already carry out similar tasks. An interviewee says:

*[one should] secure that the activities are implemented in already existing structures, so the intervention is as little cost intensive as possible.*

(Researcher)

However, the interviewees disagree about which parts of the Danish health care system is ideal for implementation of preventive health checks. Some argue for municipal health centers, others believe that general practice is the best place for preventive health checks, while some argue that a stratification of participants may contribute to optimizing the use of the health system resources – for example, by directing citizens to the municipality or general practice depending on the citizens' preventive needs.

Regardless of where and how an intervention is implemented, the working relationship between those who deliver the activities influence the success of the implementation and overall outcome. A satisfying working relationship implies that the parties are familiar with each other’s tasks and roles, which enables them to draw on each other’s resources and competences [78]. An interviewee, who evaluated a preventive health check intervention that made use of both general practice and a local municipality health center, revealed that some of the participating GPs, who had limited knowledge about the municipality’s preventive activities, were not confident that the activities met the needs of the socio-economic disadvantaged target group. Thus, they rarely referred these patients to the municipality.

*There were some GPs who did not believe that the municipality could handle the patients, so they did not refer those patients. Because ‘why should I refer my patients to something I do not believe in myself?’. Part of it was lack of knowledge about the existing activities, and some did not believe the municipality could carry out the task.*

(Researcher)

A systematic review found that the most frequent barrier for effective implementation of preventive health check interventions in the primary health care is lack of collaboration with other organizations who also take part in delivering intervention activities [25]. The interviewees in this realist synthesis suggest professional back-and-forth dialogues to increase knowledge about workflows, tasks, and competencies which are fundamental for constructive collaboration.

### Theme 6: Shared understanding of the intervention

With this theme we show how a common understanding of an intervention’s aim and design is essential for successful implementation and evaluation.

Uncertainty about the aim of an intervention appears to be a significant challenge for both the implementation and evaluation of an intervention. Some interviewees reveal having faced different interpretations of the primary purpose between the intervention parties, and thus several perceptions of whether and when success was achieved. Thus, ambiguity about the aim challenges defining a shared understanding of intervention success, which additionally affects decisions about implementation. Shared understanding supports that the intervention is implemented according to plan, because the involved parties understand the connection between the aim, implementation and expected outcome. Difficulties of demonstrating effect on long-term outcomes such as mortality is revealed in both the qualitative empirical material and the literature [10, 102]. During interviews and workshops, the participants suggested proxy outcomes rather than long term outcomes and specifically suggested measuring effect on quality of life, participant satisfaction and socio-economic effect. Concurrently, participation in itself should not be a criterion for success, because participation in itself does not lead to changes in health behaviour according to both the interviewees and workshop participants.

### Theme 7: Unintended side effects

The realist synthesis approach has allowed for a broad focus on the outcomes that emerge from the activation of mechanisms in different contexts. This has provided attention to outcomes beyond the intended and assumed outcomes of preventive health checks. In the following section we present unintended outcomes identified in the data material and argue that these should be taken into account in future preventive health check interventions.

Interviewees and workshop participants raise concerns about the risk of stigmatization in targeted preventive health checks, especially in interventions where the target group is selected based on social factors such as residence and educational level. The literature identified in this synthesis only briefly touches upon stigmatization.

Some studies point out the risk of increasing inequality in health [24, 51, 81, 103]. According to these studies, socially disadvantaged people, who are identified as those who benefit most from preventive health checks because of higher prevalence of adverse health status and behaviour, are least likely to participate. Some interviewees express reluctance about targeted preventive health checks. They argue that despite targeting an intervention towards high-risk groups participants will always be the most resourceful people within that group. Thus, regardless of the overall aim, it is essential to consider the risk of increasing inequality in health.

Another concern presented in several interviews is whether preventive health checks lead to overdiagnosis and -medication. Moreover, prevention is mentioned to contribute to push the boundaries of illness and the need for medication:

*We must not underestimate that some have great interest in screening, and health checks. And that’s the pharmaceutical industry […] So, if you consult your GP feeling healthy, you have no symptoms, he measures your blood pressure and whoops! Suddenly you might benefit from [medication]. This kind of medicalization […] That is also something that needs to be discussed when talking about screenings and health checks.*

(Researcher)

Another worry presented in a workshop was the risk that health checks could cause a sense of false security. A similarly worry is found in a qualitative study with interviews of Danish GPs [104]. Some workshop participants argue that the concept “health check” is misleading because it may give the participants an impression of guarantee of not having any illness, if the health check does not lead to further examinations or treatment. In this line, a potential risk is that participants stop reporting symptoms after a health check, because they expect everything to be fine after having their health checked recently. Consequently, this could lead to a higher mortality, which for example was found among female participants in the Danish population-based randomized lifestyle intervention Inter99 carried out in the years 1999–2006 [105]. Instead, a workshop participant suggests using words such as “diabetes screening”, which clearly illustrates the focus of the health check.

## Discussion

In this realist synthesis we find that the design and implementation of preventive health check interventions depend on the selection of a target group and the contextual circumstances. Consequently, the identified program theory is not a one-size-fits-all model, but recommendations for under which circumstances and how targeted preventive health check interventions may be expected to work based on the best available knowledge.

The findings in this study show that the success of preventive health check interventions rests on several criteria. Among other things the activities of the health check need to be accessible, recognizable, and relevant for the participants’ everyday lives. Furthermore, the activities need to be meaningful to the professionals involved in the implementation and operation of the intervention, and sufficient resources need to be allocated to the implementation process. These findings, correlate well with a recent realist review focusing on the variation in advice and support offered to NHS Health Check attendees [106]. In accordance with our findings, the realist review shows that differences in local priorities, such as workload and time, may affect the availability and accessibility of referral pathways to local ‘lifestyle services’ [106]. Moreover, the review points out that some attendees may face external challenges related to their individual circumstances which affect their capacity to engage in lifestyle changes [106]. In our study we came across a similar finding. Thus, while this realist synthesis indicated that people or groups who are at increased risk of chronic disease potentially may benefit the most from preventive health checks, the findings additionally showed that those identified as being at increased risk often do not have the resources or opportunity to participate. Identifying a target group, that both benefits from attending the preventive health checks and has the resources to participate, poses a challenge for preventive health check interventions. At the same time, this challenge illustrates the importance of designing the recruitment and intervention activities according to the particular life situation and needs of the target group.

The findings of the realist synthesis point to the necessity of conducting a thorough needs assessments when planning and developing future preventive health check interventions. Needs assessments are recommended for the development of complex interventions and are used to explore the needs of the target group, community, and/or relevant stakeholders [107, 108]. Needs can range from prevention and treatment to, for example, social needs connected to the target group’s everyday life [28]. The aim of a needs assessment is to prioritize, compare, and balance different kinds of needs. Identification of different needs will further help clarify the overall aim, potential outcomes, and success criteria, which, according to the data material, facilitate and strengthen the implementation and operation of the intervention.

### Strengths and limitations

Applying the realist synthesis as a methodological approach accommodates the complex setup of preventive health check interventions. Nevertheless, the different contextual circumstances framing the preventive health interventions under study challenged linking the identified mechanisms in the qualitative studies with the outcomes identified in the quantitative studies. Among other things, contextual circumstances are rarely described in detail in quantitative studies, and even though qualitative studies include contextual circumstances, these studies cannot illuminate all parts of the program theory. To this end, we conducted interviews and workshops which provided insights into contextual conditions of Danish preventive health checks. However, there are elements of preventive health checks that we could not get access to through the literature nor interviews or workshops, due to the complexity of this field of research and the various designs. Furthermore, international differences in healthcare systems calls for attention to the generalizability of some of the results.

The iterative phase design of this study, though, provided the opportunity to continuously test, refine and add nuance to the hypotheses. For example, the interplay between the context *short geographical distance/proper public transport* and the mechanism *accessibility* was found in the first phase and confirmed throughout the other three phases. Through the ongoing testing, the hypothesis was refined to not only provide recommendation regarding the maximal distance to the health check, but also include attention to the specific target group perceptions of a tolerable geographical distance. In this way, the different methods and iterative analysis of the data material contributed to strengthening the overall program theory.

### Unanswered questions and future research

In the field of prevention, there is an ongoing discussion about systematic versus opportunistic health checks [19, 109]. As part of this discussion, economic considerations play a central role. In recent years, a number of studies have examined the cost-effectiveness of different targeted and general preventive health check interventions, and some are compared to usual care, thus the opportunistic approach. While one study shows that opportunistic screening in general practice is cheaper, but just as effective in identifying people with unrecognized T2D as a stepwise screening procedure identifying risk by use of a questionnaire [110], most studies indicate that the implementation of targeted approaches are more cost-effective than usual care [111–113]. Some studies demonstrate that targeted preventive interventions are more cost-effective compared to interventions offered to the general population – especially if the intervention targets people with a BMI ≥ 30 kg/m2 [50, 114]. This realist synthesis does not answer questions regarding the cost-effectiveness of targeted preventive health checks, but we suggest further studies of cost-effectiveness in relation to different high-risk target groups to help answer for whom targeted preventive health checks work.

Drawing on the realist research approach enabled us to pay attention to both intended and unintended consequences of preventive health checks. Generally, attention to unintended consequences of preventive health checks is rare in the literature and our findings of unintended side effects are primarily based on the qualitative examinations in the study [115]. However, knowledge about unintended consequences contributes important information and recommendations for future research in this field, both in terms of strengthening the implementation of health checks and in relation to the knowledge gap that exist in the literature. Including attention to unintended consequences of preventive health checks, might help rethink any of the activities involved in order to reduce negative unintended side effects or enable researchers to explore the unintended side effects and in this way hinder or enhance outcomes [116]. We suggest that future preventive health checks incorporate attention to unintended side effects in order to fully understand the impact of the health checks.

## Conclusion

This study illustrates factors that influence whether targeted preventive health checks lead to intended effects. Because design and implementation are dependent on the target group and the contextual circumstances, this realist synthesis does not provide instructions or recommendations for the specific design or implementation for future interventions. On the contrary, the results indicate that a one-size-fits-all model should be abandoned, and that intervention activities and implementation depend on for whom and under which circumstances the intervention is initiated.

Because the results show that a thorough needs assessment is pivotal before deciding on any recruitment, intervention, and implementation strategies, the selection of a target group its specific needs, should form the basis for decisions about where and how preventive health checks are implemented.

### Supplementary Information

**Additional file 1.** 

**Additional file 2.** 

**Additional file 3.** 

**Additional file 4.**

## Data Availability

Due to the sensitive nature of the data generated and the possibility of identification of individuals, the data generated and/or analyzed during the current study are not publicly available. Please contact Marie Broholm-Jørgensen to request data from this study.

## Abbreviations

CMO: Context, Mechanisms and Outcomes
GP: General Practitioner
T2D: Type-2-diabetes
