# Supplementary material for: Exploring targeted preventive health check interventions – a realist synthesis
Source: BMC Public Health. 2023 Oct 5;23:1928. doi: 10.1186/s12889-023-16861-8 (PMC10557298; doi:10.1186/s12889-023-16861-8)
Supplement: Supplementary file 1 — Additional file 1. [file 12889_2023_16861_MOESM1_ESM.pdf]

Supplementary material 1:

Search terms for phase 1.

Databases: Embase, PsychINFO, Cochrane Central Database and Global

Health Latest search carried out on May 21st 2021.

Search Terms:

Intervention AND condition AND setting AND type of study AND target group country AND targeted

#	Searches	Results
1	Health check.mp.	5218
2	Healthcheck.mp.	30
3	1 or 2	5244
4	health check*.mp.	9190
5	3 or 4	9213
6	prevention/ or primary prevention/ or prophylaxis/ or secondary prevention/ or smoking prevention/	474104
7	Preven*.mp.	3104743
8	6 or 7	3159371
9	behavio?r advi*.mp.	50
10	behavio?ral advi*.mp. [mp=title, abstract, heading word, drug trade name, original title, device manufacturer, drug manufacturer, device trade name, keyword, floating subheading word, candidate term word]	65
11	behavio?r modification*.mp.	11194
12	behavio?ral modification*.mp.	2683
13	behavior modification/	8310
14	9 or 10 or 11 or 12 or 13	13451
15	lifestyle modification/	42060
16	lifestyle modification*.mp.	46529
17	lifestyle intervention*.mp.	11697
18	lifestyle advi*.mp.	1583
19	15 or 16 or 17 or 18	55132
20	motivational interviewing/	5483
21	motivational interview*.mp.	8413
22	20 or 21	8413
23	screening program*.mp.	46315
24	health dialogue*.mp.	94
25	prevent* program*.mp. [mp=title, abstract, heading word, drug trade name, original title, device manufacturer, drug manufacturer, device trade name, keyword, floating subheading word, candidate term word]	33953
26	5 or 8 or 14 or 19 or 22 or 23 or 24 or 25	3252118
27	exp non communicable disease/di, pc [Diagnosis, Prevention]	544
28	non communicable disease*.mp. [mp=title, abstract, heading word, drug trade name, original title, device manufacturer, drug manufacturer, device trade name, keyword, floating subheading word, candidate term word]	15171

29	ncd.mp. [mp=title, abstract, heading word, drug trade name, original title, device manufacturer, drug manufacturer, device trade name, keyword, floating subheading word, candidate term word]	4197
30	ncds.mp. [mp=title, abstract, heading word, drug trade name, original title, device manufacturer, drug manufacturer, device trade name, keyword, floating subheading word, candidate term word]	3976
31	exp chronic disease/di, pc [Diagnosis, Prevention]	6858
32	chronic disease*.mp. [mp=title, abstract, heading word, drug trade name, original title, device manufacturer, drug manufacturer, device trade name, keyword, floating subheading word, candidate term word]	265210
33	chronic illness.mp. [mp=title, abstract, heading word, drug trade name, original title, device manufacturer, drug manufacturer, device trade name, keyword, floating subheading word, candidate term word]	19275
34	chronic illnesses.mp. [mp=title, abstract, heading word, drug trade name, original title, device manufacturer, drug manufacturer, device trade name, keyword, floating subheading word, candidate term word]	7129
35	27 or 28 or 29 or 30 or 31 or 32 or 33 or 34	292390
36	chronic obstructive lung disease/di, pc [Diagnosis, Prevention]	11346
37	chronic obstructive lung disease.mp.	147200
38	copd.mp. [mp=title, abstract, heading word, drug trade name, original title, device manufacturer, drug manufacturer, device trade name, keyword, floating subheading word, candidate term word]	96101
39	non insulin dependent diabetes mellitus/di, pc [Diagnosis, Prevention]	17686
40	non insulin dependent diabetes mellitus.mp. [mp=title, abstract, heading word, drug trade name, original title, device manufacturer, drug manufacturer, device trade name, keyword, floating subheading word, candidate term word]	279017
41	t2dm.mp.	42053
42	type 2 diabetes.mp. [mp=title, abstract, heading word, drug trade name, original title, device manufacturer, drug manufacturer, device trade name, keyword, floating subheading word, candidate term word]	214692
43	cardiovascular disease/di, pc [Diagnosis, Prevention]	41114
44	cardiovascular disease*.mp. [mp=title, abstract, heading word, drug trade name, original title, device manufacturer, drug manufacturer, device trade name, keyword, floating subheading word, candidate term word]	441553
45	cholesterol/ec [Endogenous Compound]	103441
46	36 or 37 or 38 or 39 or 40 or 41 or 42 or 43 or 44 or 45	959069
47	35 or 46	1221470
48	primary medical care/	113945
49	Primary care.mp. [mp=title, abstract, heading word, drug trade name, original title, device manufacturer, drug manufacturer, device trade name, keyword, floating subheading word, candidate term word]	172351
50	primary health care/	70736
51	Primary health care.mp. [mp=title, abstract, heading word, drug trade name, original title, device manufacturer, drug manufacturer, device trade name, keyword, floating subheading word, candidate term word]	83933
52	Primary healthcare.mp.	8599
53	general practice/	87926
54	general practice.mp. [mp=title, abstract, heading word, drug trade name, original title, device manufacturer, drug manufacturer, device trade name, keyword, floating subheading word, candidate term word]	105697
55	family practice.mp.	10252

56	family doctor.mp.	4511
57	family medicine/	11865
58	family medicine.mp. [mp=title, abstract, heading word, drug trade name, original title, device manufacturer, drug manufacturer, device trade name, keyword, floating subheading word, candidate term word]	19413
59	physician/	329032
60	physician.mp. [mp=title, abstract, heading word, drug trade name, original title, device manufacturer, drug manufacturer, device trade name, keyword, floating subheading word, candidate term word]	579515
61	general practitioner/	110325
62	general practitioner.mp. [mp=title, abstract, heading word, drug trade name, original title, device manufacturer, drug manufacturer, device trade name, keyword, floating subheading word, candidate term word]	122301
63	health professional.mp. [mp=title, abstract, heading word, drug trade name, original title, device manufacturer, drug manufacturer, device trade name, keyword, floating subheading word, candidate term word]	12338
64	health practitioner/	58450
65	practitioner.mp.	234771
66	preventive medicine/	29397
67	preventive medicine.mp. [mp=title, abstract, heading word, drug trade name, original title, device manufacturer, drug manufacturer, device trade name, keyword, floating subheading word, candidate term word]	36625
68	municipal*.mp.	57782
69	48 or 49 or 50 or 51 or 52 or 53 or 54 or 55 or 56 or 57 or 58 or 59 or 60 or 61 or 62 or 63 or 64 or 65 or 66 or 67 or 68	1099184
70	exp review/	2849696
71	(literature adj3 review$).ti,ab.	404878
72	exp meta analysis/	217843
73	exp "systematic review"/	297177
74	70 or 71 or 72 or 73	3177249
75	(medline or medlars or embase or pubmed or cinahl or amed or psychlit or psyclit or psychinfo or psycinfo or scisearch or cochrane).ti,ab.	335047
76	RETRACTED ARTICLE/	11437
77	75 or 76	346104
78	74 and 77	265332
79	(systematic$ adj2 (review$ or overview)).ti,ab.	274612
80	(meta?anal$ or meta anal$ or meta-anal$ or metaanal$ or metanal$).ti,ab.	264501
81	78 or 79 or 80	504302
82	at risk.mp.	276072
83	increased risk.mp.	376796
84	high risk.mp.	652992
85	exp high risk behavior/	29331
86	risk factor*.mp. [mp=title, abstract, heading word, drug trade name, original title, device manufacturer, drug manufacturer, device trade name, keyword, floating subheading word, candidate term word]	1484568
87	risk factor/di [Diagnosis]	1
88	high risk patient/	142521
89	high risk patient*.mp.	168316
90	82 or 83 or 84 or 85 or 86 or 87 or 88 or 89	2402363
91	26 and 47 and 69 and 81 and 90	397

92	targeted.mp. [mp=title, abstract, heading word, drug trade name, original title, device manufacturer, drug manufacturer, device trade name, keyword, floating subheading word, candidate term word]	535654
93	targeting.mp. [mp=title, abstract, heading word, drug trade name, original title, device manufacturer, drug manufacturer, device trade name, keyword, floating subheading word, candidate term word]	646793
94	specific.mp.	3382028
95	tailored.mp.	88425
96	tailoring.mp. [mp=title, abstract, heading word, drug trade name, original title, device manufacturer, drug manufacturer, device trade name, keyword, floating subheading word, candidate term word]	22987
97	selective.mp.	683059
98	selected.mp.	1087006
99	92 or 93 or 94 or 95 or 96 or 97 or 98	5699511
100	26 and 47 and 69 and 81 and 90 and 99	135

Search terms for phase 3.

Databases: Embase PsychINFO, Cochrane Central Database and Global Health

Latest search carried out on December 8th 2021.

Search terms:

Intervention AND setting AND targeted AND target group AND type of study AND country

#	Searches	Results
1	Health check.mp.	5639
2	Healthcheck.mp.	30
3	1 or 2	5664
4	health check*.mp.	10023
5	3 or 4	10046
6	prevention/ or primary prevention/ or prophylaxis/ or secondary prevention/ or smoking prevention/	491374
7	Preven*.mp.	3252255
8	6 or 7	3310538
9	behavio?r advi*.mp.	54
10	behavio?ral advi*.mp. [mp=title, abstract, heading word, drug trade name, original title, device manufacturer, drug manufacturer, device trade name, keyword heading word, floating subheading word, candidate term word]	73
11	behavio?r modification*.mp.	11276
12	behavio?ral modification*.mp.	2815
13	behavior modification/	8339
14	9 or 10 or 11 or 12 or 13	13672
15	lifestyle modification/	45868
16	lifestyle modification*.mp.	50737
17	lifestyle intervention*.mp.	12900
18	lifestyle advi*.mp.	1680
19	15 or 16 or 17 or 18	60218
20	motivational interviewing/	6072
21	motivational interview*.mp.	9148
22	20 or 21	9148

23	screening program*.mp.	48801
24	health dialogue*.mp.	101
25	3 or 5 or 8 or 14 or 19 or 22 or 23 or 24	3410183
26	primary medical care/	119650
27	Primary care.mp. [mp=title, abstract, heading word, drug trade name, original title, device manufacturer, drug manufacturer, device trade name, keyword heading word, floating subheading word, candidate term word]	184126
28	primary health care/	74807
29	Primary health care.mp. [mp=title, abstract, heading word, drug trade name, original title, device manufacturer, drug manufacturer, device trade name, keyword heading word, floating subheading word, candidate term word]	88934
30	Primary healthcare.mp.	9885
31	general practice/	89090
32	general practice.mp. [mp=title, abstract, heading word, drug trade name, original title, device manufacturer, drug manufacturer, device trade name, keyword heading word, floating subheading word, candidate term word]	107270
33	family practice.mp.	10358
34	family doctor.mp.	4630
35	family medicine/	12365
36	family medicine.mp. [mp=title, abstract, heading word, drug trade name, original title, device manufacturer, drug manufacturer, device trade name, keyword heading word, floating subheading word, candidate term word]	20712
37	physician/	345797
38	physician.mp. [mp=title, abstract, heading word, drug trade name, original title, device manufacturer, drug manufacturer, device trade name, keyword heading word, floating subheading word, candidate term word]	605132
39	general practitioner/	114625
40	general practitioner.mp. [mp=title, abstract, heading word, drug trade name, original title, device manufacturer, drug manufacturer, device trade name, keyword heading word, floating subheading word, candidate term word]	126854
41	health professional.mp. [mp=title, abstract, heading word, drug trade name, original title, device manufacturer, drug manufacturer, device trade name, keyword heading word, floating subheading word, candidate term word]	13284
42	health practitioner/	59255
43	practitioner.mp.	242892
44	preventive medicine/	29631
45	preventive medicine.mp. [mp=title, abstract, heading word, drug trade name, original title, device manufacturer, drug manufacturer, device trade name, keyword heading word, floating subheading word, candidate term word]	37341
46	municipal*.mp.	62096
47	26 or 27 or 28 or 29 or 30 or 31 or 32 or 33 or 34 or 35 or 36 or 37 or 38 or 39 or 40 or 41 or 42 or 43 or 44 or 45 or 46	1148427
48	targeted.mp. [mp=title, abstract, heading word, drug trade name, original title, device manufacturer, drug manufacturer, device trade name, keyword heading word, floating subheading word, candidate term word]	595588

49	targeting.mp. [mp=title, abstract, heading word, drug trade name, original title, device manufacturer, drug manufacturer, device trade name, keyword heading word, floating subheading word, candidate term word]	705331
50	specific.mp.	3560268
51	tailored.mp.	98957
52	tailoring.mp. [mp=title, abstract, heading word, drug trade name, original title, device manufacturer, drug manufacturer, device trade name, keyword heading word, floating subheading word, candidate term word]	25553
53	selective.mp.	710659
54	selected.mp.	1158613
55	adjust*.mp. [mp=title, abstract, heading word, drug trade name, original title, device manufacturer, drug manufacturer, device trade name, keyword heading word, floating subheading word, candidate term word]	1079315
56	flexib*.mp. [mp=title, abstract, heading word, drug trade name, original title, device manufacturer, drug manufacturer, device trade name, keyword heading word, floating subheading word, candidate term word]	248103
57	personali*.mp. [mp=title, abstract, heading word, drug trade name, original title, device manufacturer, drug manufacturer, device trade name, keyword heading word, floating subheading word, candidate term word]	314272
58	48 or 49 or 50 or 51 or 52 or 53 or 54 or 55 or 56 or 57	7325332
59	at risk.mp.	296269
60	increased risk.mp.	408534
61	high risk.mp.	705380
62	exp high risk behavior/	30621
63	risk factor*.mp. [mp=title, abstract, heading word, drug trade name, original title, device manufacturer, drug manufacturer, device trade name, keyword heading word, floating subheading word, candidate term word]	1604054
64	risk factor/di [Diagnosis]	1
65	high risk patient/	152698
66	high risk patient*.mp.	180348
67	high risk population/	140163
68	minority group/	17306
69	ethnic group/	79611
70	ethnic minorit*.mp. [mp=title, abstract, heading word, drug trade name, original title, device manufacturer, drug manufacturer, device trade name, keyword heading word, floating subheading word, candidate term word]	17895
71	racial group*.mp. [mp=title, abstract, heading word, drug trade name, original title, device manufacturer, drug manufacturer, device trade name, keyword heading word, floating subheading word, candidate term word]	10969
72	unemployment/	24821
73	unemploy*.mp. [mp=title, abstract, heading word, drug trade name, original title, device manufacturer, drug manufacturer, device trade name, keyword heading word, floating subheading word, candidate term word]	36167
74	men.mp.	830620
75	rural population/ or rural area/	116762

76	rural*.mp. [mp=title, abstract, heading word, drug trade name, original title, device manufacturer, drug manufacturer, device trade name, keyword heading word, floating subheading word, candidate term word]	232570
77	outskirts.mp. [mp=title, abstract, heading word, drug trade name, original title, device manufacturer, drug manufacturer, device trade name, keyword heading word, floating subheading word, candidate term word]	1031
78	deprived.mp.	39307
79	poor.mp.	999860
80	poverty.mp. or poverty/	67238
81	disadvantaged.mp.	18706
82	socioeconomic.mp. or socioeconomics/	256047
83	uneducated.mp.	1221
84	59 or 60 or 61 or 62 or 63 or 64 or 65 or 66 or 67 or 68 or 69 or 70 or 71 or 72 or 73 or 74 or 75 or 76 or 77 or 78 or 79 or 80 or 81 or 82 or 83	4542744
85	qualitative research/	100915
86	qualitative research.mp. [mp=title, abstract, heading word, drug trade name, original title, device manufacturer, drug manufacturer, device trade name, keyword heading word, floating subheading word, candidate term word]	113617
87	qualitative study.mp.	61007
88	ethnographic research/	1030
89	ethnography/	3292
90	Ethnograph*.mp.	15086
91	participant observation/	5345
92	participant observation.mp. [mp=title, abstract, heading word, drug trade name, original title, device manufacturer, drug manufacturer, device trade name, keyword heading word, floating subheading word, candidate term word]	9269
93	qualitative methods.mp.	10272
94	85 or 86 or 87 or 88 or 89 or 90 or 91 or 92 or 93	153246
95	25 and 47 and 58 and 84	20033
96	94 and 95	572
97	denmark.mp. or Denmark/	89512
98	danish.mp.	43860
99	United Kingdom/ or England/	463894
100	england.mp. [mp=title, abstract, heading word, drug trade name, original title, device manufacturer, drug manufacturer, device trade name, keyword heading word, floating subheading word, candidate term word]	123144
101	United Kingdom.mp. [mp=title, abstract, heading word, drug trade name, original title, device manufacturer, drug manufacturer, device trade name, keyword heading word, floating subheading word, candidate term word]	576206
102	british.mp.	125301
103	english.mp.	159267
104	great britain.mp. or Great Britain/	22183

105	dutch.mp.	61901
106	the netherlands.mp. or Netherlands/	116028
107	holland.mp. or Netherlands/	94510
108	Scandinavia/ or scandinavia*.mp.	18438
109	nordic countries.mp.	3399
110	norway.mp. or Norway/	72684
111	norwegian.mp. or "Norwegian (people)"/	27577
112	sweden.mp. or Sweden/	140120
113	swedish.mp.	57569
114	Finland/	42293
115	finland.mp. [mp=title, abstract, heading word, drug trade name, original title, device manufacturer, drug manufacturer, device trade name, keyword heading word, floating subheading word, candidate term word]	59640
116	finnish.mp.	25417
117	greenland.mp. or Greenland/	4914
118	greenlandic.mp.	591
119	iceland.mp. or Iceland/	8993
120	icelandic.mp.	3291
121	Faroe Islands/ or the faroe islands.mp.	698
122	faroese.mp. or Faroese/	265
123	faroe islands.mp. [mp=title, abstract, heading word, drug trade name, original title, device manufacturer, drug manufacturer, device trade name, keyword heading word, floating subheading word, candidate term word]	711
124	97 or 98 or 99 or 100 or 101 or 102 or 103 or 104 or 105 or 106 or 107 or 108 or 109 or 110 or 111 or 112 or 113 or 114 or 115 or 116 or 117 or 118 or 119 or 120 or 121 or 122 or 123	1456461
125	96 and 124	135
