# Supplementary material for: Exploring targeted preventive health check interventions – a realist synthesis
Source: BMC Public Health. 2023 Oct 5;23:1928. doi: 10.1186/s12889-023-16861-8 (PMC10557298; doi:10.1186/s12889-023-16861-8)
Supplement: Supplementary file 2 — Additional file 2. [file 12889_2023_16861_MOESM2_ESM.docx]

**Supplementary file 2:**

This file contains three program theories developed during the realist synthesis.

***The initial program theory (phase 1):***

*CMO 1: Targeted*

People with poor mental health (anxiety and worries), increased risk of chronic disease (due to lifestyle risk factors and/or genetic predisposition), men, people with low socioeconomic status, people living in areas characterized with low income, low level of education and high unemployment hold the greatest potential for reducing the risk through changes in health behavior.

*CMO 2: Experience of relevance in everyday life*

People who experience health-related concerns, who are genetic predisposed or people with a high level of health literacy are motivated to participate in preventive health checks and change adverse health behavior due to participation in a health check.

*CMO 3: Personal and frequent communication*

Personal and frequent communication (e.g., patient-centered goals, follow-up consultations, phone calls and e-mails from the GP etc.) motivates participants to attending and prevents negative effects of psychosocial outcomes

*CMO 4: Accessibility*

Existing system and (health) services as well as resources provided in the local cultural context (language, level of health literacy, geographical distance etc.) increase the experience of accessibility and thus the participation.

*CMO 5: Implementation support*

Time pressure, staff, finances (including financial compensation), political and managerial support, IT, (upgraded) skills influence how those who deliver the health check spend their time and resources.

*CMO 6: perspectives and knowledge about prevention*

The attitude towards and knowledge about the benefit of prevention of the people who deliver the health checks activates and influence the implementation of preventive health checks.

***The tested and refined program theory (phase 2):***

*CMO 1: Target group*

1. People at high risk, e.g., poor mental health, risk of chronic disease (due to lifestyle risk factors and/or known illness in the immediate family), men, ethnic minorities, people with low socioeconomic status, people living in areas characterized with low income, low level of education and high unemployment hold the greatest potential for reducing the risk through changes in health behavior.
2. People at high risk, such as poor mental health, people with low socioeconomic status, people living in areas characterized with low income, low level of education and high unemployment have less surplus of mental resources to respond to an invitation to a preventive health check, participate or change adverse health behavior.

*CMO 2: Recruitment and participation*

1. Experience of relevance in everyday life

People who experience health-related concerns, people who experience symptoms of disease, who are genetic predisposed or people with a high level of health literacy find that preventive health checks are relevant in their everyday lives and are thus motivated to participate in preventive health checks and change adverse health behavior due to participation in a health check and change adverse health behavior.

1. Accessibility

A focus on accessibility (geographical distance, language, easy booking processes and modification of the preventive activities according to the needs of the participants) and recognition (existing systems, (health) services as well as resources provided in the local cultural context and that the GP appear as the sender) in the invitation to and the implementation of the preventive health check will increase recruitment and participation.

*CMO 3: Implementation support*

Time pressure, staff, finances (including financial compensation), political and managerial support, familiarity and trust among the people who are involved in delivering the preventive health checks, the attitude towards and knowledge about the benefit of prevention as well as the division of preventive tasks in relation to the existing primary health care sector influence how those who deliver the health check spend their time and resources.

*CMO 4: Sustainability*

Preventive health checks that are implemented in an already existing systems or services have an easier start-up, have an increase in sustainability after the project period ends, have an optimal use of the resources in the health care sector and an increase in participants’ experience of accessibility and thus participation.

*CMO 5: Shared understanding*

Consistent and clear definitions of the aim, primary outcome and success criteria of the preventive health checks at all levels facilitate implementation in the proper section of the health care system and ease goal setting for implementation and evaluation.

*Unintended side effects*

- Increase of collaboration in the primary health sector
- Increase of knowledge about the patient in general practice consultations
- Risk of stigmatization
- Risk of over-diagnosis and -medication
- Risk of sickening participants

***The tested and refined program theory (phase 3):***

*Target group:*

If preventive health checks target people at high risk, then the greatest potential for reducing the risk is achieved.

People at risk are:

- Men
- Ethnic minorities
- people with low level of education and/or high unemployment
- People with poor mental health
- People with risk of chronic disease (based on algorithms)
- People with mental and physical disabilities

If preventive health checks target people who are at high risk and who have a low degree of resources and flexibility in their everyday lives are less likely to respond to the invitation, participate in preventive health checks and change adverse health behavior.

People at high risk and who have a low degree of resources and flexibility are:

- People living in local areas with low socioeconomic status
- People with poor mental health
- people with low level of education and/or high unemployment

*CMO configurations:*

| **Theme** | **Context** | **Mechanism** | **Outcome** |
| --- | --- | --- | --- |
| **Recruitment and participation** | - Illness among relatives or friends - Health-related concerns - Experiences of symptoms of disease - high level of health literacy | - Experience of relevance | - Increase in participation - Change of adverse health behaviour |
|  | - Short geographical distance/proper public transport - Targeted language - Simple booking procedures - Activities accommodated to the needs of the target group | - Experience of accessibility | - Increase in participation |
|  | - Use of the existing systems in the health care system - (health) services as well as resources provided in the local cultural context - The GP appear is the sender of the invitation | - Trust - A sense of security - Familiarity - Authority | - Increase in participation |
| **Implementation support** | - Time - Staff - Finances - Shared understanding of the division of tasks - Political and managerial support - familiarity and trust among the people who are involved in delivering the preventive health checks - attitude towards and knowledge about the benefit of prevention | - Motivation - Relevance | - Ease of implementation - Sustainability - optimal use of the resources in the health care sector - Increase in time and resources spend |
| **Shared understanding** | - Clear definition of aim - Clear definition of outcome - Clear definition of success criteria | - Shared understanding among professionals delivering the preventive health checks | - Strengthened implementation and evaluation |

*Unintended side effects*

- Increase of collaboration in the primary health sector
- Increase of knowledge about the patient in general practice consultations
- Risk of stigmatization
- Risk of over-diagnosis and -medication
- Risk of sickening participants
