# Supplementary material for: Exploring targeted preventive health check interventions – a realist synthesis
Source: BMC Public Health. 2023 Oct 5;23:1928. doi: 10.1186/s12889-023-16861-8 (PMC10557298; doi:10.1186/s12889-023-16861-8)
Supplement: Supplementary file 3 — Additional file 3. [file 12889_2023_16861_MOESM3_ESM.pdf]

Supplementary file 3:

RAMESES checklist

List of items when reporting a realist synthesis

Section		Description	Page number
1	Title	In the title, identify the document as a realist synthesis or review	1
2	Abstract	While acknowledging publication requirements and house style, abstracts should ideally contain brief details of: the study's background, review question or objectives; search strategy; methods of selection, appraisal, analysis and synthesis of sources; main results; and implications for practice.	2
**Introduction**			
3	Rationale	Explain why the review is needed and what it is likely to contribute to existing understanding of the topic area.	4
4	Objectives	State the objective(s) of the review and/or the review question(s). Define and provide a rationale for the focus of the review.	4-5
**Methods**			
5	Changes	Any changes made to the review process that was initially planned should be briefly described and justified.	N/A
6	Rationale for realist	Explain why realist synthesis was considered the most appropriate method to use	6-7
7	Scoping	Describe and justify the initial process of exploratory scoping of the literature.	7, 11
8	Search	While considering specific requirements of the journal or other publication outlet, state and provide a rationale for how the iterative searching was done. Provide details on all the sources accessed for information in the review. Where searching in electronic databases has taken place, the details should include, for example, name of database, search terms, dates of coverage	7-12, Supplementary file 1

Section		Description	Page number
		and date last searched. If individuals familiar with the relevant literature and/or topic area were contacted, indicate how they were identified and selected	
9	Selection and appraisal	Explain how judgements were made about including and excluding data from documents and justify these.	7-10 figure 2 and figure 3
10	Extraction	Describe and explain which data or information were extracted from the included documents and justify this selection.	7-10, figure 2 and figure 3
11	Analysis and synthesis	Describe the analysis and synthesis processes in detail. This section should include information on the constructs analyzed and describe the analytic process.	11-12 and Supplementary file 2
**Results**			
12	Flow diagram	Provide details on the number of documents assessed for eligibility and included in the review with reasons for exclusion at each stage as well as an indication of their source of origin (for example, from searching databases, reference lists and so on). You may consider using the example templates (which are likely to need modification to suit the data) that are provided.	13, figure 2 and figure 3.
13	Document characteristics	Provide information on the characteristics of the documents included in the review.	13
14	Main findings	Present the key findings with a specific focus on theory building and testing.	14 and 15-28
**Discussion and conclusion**			
15	Summary	Summarize the main findings, taking into account the review's objective(s), research question(s), focus and intended audience(s).	29
16	Strengths and weaknesses	Discuss both the strengths of the review and its limitations. These should include (but need not be restricted to) (a) consideration of all the steps in the review process and (b) comment on the overall strength of evidence supporting the explanatory insights which emerged. The limitations identified may point to areas where further work is needed.	30-31
17	Comparison	Where applicable, compare and contrast the review's findings with the existing literature (for example, other reviews) on the same topic.	29-30

Section		Description	Page number
18	Conclusion	List the main implications of the findings and place these in the context of other relevant literature. If appropriate, offer recommendations for policy and practice.	32
19	Funding	Provide details of funding source (if any) for the review, the role played by the funder (if any) and any conflicts of interests of the reviewers.	33-34
