# Supplementary material for: Exploring targeted preventive health check interventions – a realist synthesis
Source: BMC Public Health. 2023 Oct 5;23:1928. doi: 10.1186/s12889-023-16861-8 (PMC10557298; doi:10.1186/s12889-023-16861-8)
Supplement: Supplementary file 4 — Additional file 4. [file 12889_2023_16861_MOESM4_ESM.docx]

**Supplementary file 4:**

A detailed table of alle included studies with characteristics.

| **Phase 1: Literature review of systematic reviews and meta-analyses** | | |
| --- | --- | --- |
| Author/year | Aim | Main results |
| Taggart J. et al. *A systematic review of interventions in primary care to improve health literacy for chronic disease behavioral risk factors*. 2012. | To evaluate the effectiveness of interventions used in primary care to improve health literacy for change in smoking, nutrition, alcohol, physical activity and weight (SNAPW). | 52 studies were included. Many different intervention types and settings were associated with change in health literacy (73% of all studies) and change in SNAPW (75% of studies). More low intensity interventions reported significant positive outcomes for SNAPW (43% of studies) compared with high intensity interventions (33% of studies). More interventions in primary health care than the community were effective in supporting smoking cessation whereas the reverse was true for diet and physical activity interventions. |
| Cheong A.T., Liew S.M., Khoo E.M., Mohd Zaidi N.F., Chinna K. Are interventions to increase the uptake of screening for cardiovascular disease risk factors effective? A systematic review and meta-analysis. BMC Fam Pract. 2017 Jan 17;18(1):4. doi: 10.1186/s12875- 016-0579-8. PMID: 28095788; PMCID: PMC5240221. | to determine the effectiveness of existing intervention strategies to increase uptake of CVD risk factors screening | 21 studies were included in the meta-analysis. The risk of bias for randomization was low to medium in the randomized controlled trials, except for one, and high in the non-randomized trials. Two analyses were performed; optimistic (using the highest effect sizes) and pessimistic (using the lowest effect sizes). Overall, interventions were shown to increase the uptake of screening for CVD risk factors (RR 1.443; 95% CI 1.264 to 1.648 for pessimistic analysis and RR 1.680; 95% CI 1.420 to 1.988 for optimistic analysis). Effective interventions that increased screening participation included: use of physician reminders (RR ranged between 1.392; 95% CI 1.192 to 1.625, and 1.471; 95% CI 1.304 to 1. 660), use of dedicated personnel (RR ranged between 1.510; 95% CI 1.014 to 2.247, and 2.536; 95% CI 1.297 to 4.960) and provision of financial incentives for screening (RR 1.462; 95% CI 1.068 to 2.000). Meta-regression analysis showed that the effect of CVD risk factors screening uptake was not associated with study design, types of population nor types of interventions |
| Zhou X., Karen R. Siegel K. R., Ng B. P., Jawanda S., Proia K. K., Zhang X., Albright A. L., Zhang P.; Cost-effectiveness of Diabetes Prevention Interventions Targeting High-risk Individuals and Whole Populations: A Systematic Review. Diabetes Care 1 July 2020; 43 (7): 1593–1616. https://doi.org/10.2337/dci20-0018 | To evaluate the cost-effectiveness (CE) of interventions to prevent type 2 diabetes (T2D) among high-risk individuals and whole populations. | The review included 39 studies: 28 on interventions targeting high-risk individuals and 11 targeting whole populations. Both lifestyle and metformin interventions in high-risk individuals were cost-effective from a health care system or a societal perspective, with median ICERs of $12,510/QALY and $17,089/QALY, respectively, compared with no intervention. Among lifestyle interventions, those that followed a Diabetes Prevention Program (DPP) curriculum had a median ICER of $6,212/QALY, while those that did not follow a DPP curriculum had a median ICER of $13,228/QALY. Compared with lifestyle interventions delivered one-on-one or by a health professional, those offered in a group setting or provided by a combination of health professionals and lay health workers had lower ICERs. Among population based interventions, taxing sugar-sweetened beverages was cost-saving from both the health care system and governmental perspectives. Evaluations of other population-based interventions including fruit and vegetable subsidies, community-based education programs, and modifications to the built environment showed inconsistent results. |
| Versteeg H, Spek V, Pedersen SS, Denollet J. Type D personality and health status in cardiovascular disease populations: a meta-analysis of prospective studies. European Journal of Preventive Cardiology. 2012;19(6):1373-1380. doi:10.1177/1741826711425338 | To examine the association between Type D personality and the individual differences in patient-reported physical and mental health status among cardiovascular patients. | Of all identified studies, ten studies met the selection criteria. The meta-analyses showed that Type D was associated with a two-fold increased odds for impaired physical health status (3035 patients, OR 1.94, 95% CI 1.49–2.52) and a 2.5-fold increased odds for impaired mental health status (2213 patients, OR 2.55, 95% CI 1.57–4.16). There was no significant heterogeneity between the studies on physical health status (Q = 12.78; p = 0.17; I2 = 29.59), but there was between those on mental health status (Q = 21.91; p = 0.003; I2 = 68.04). Subgroup analyses showed that the association between Type D and mental health status decreased yet remained significant when adjusting for baseline health status. |
| Lawlor E. R., Bradley D. T., Cupples M. E., Tully M. A. The effect of community-based interventions for cardiovascular disease secondary prevention on behavioural risk factors. Prev Med. 2018 Sep;114:24-38. doi: 10.1016/j.ypmed.2018.05.019. Epub 2018 May 24. PMID: 29802876. | To assess the effectiveness of secondary prevention cardiovascular risk reduction programmes delivered in venues situated within the community on modification of behavioural risk factors | Meta-analyses identified increased steps/week (Mean Difference (MD): 7480; 95% CI 1,940, 13,020) and minutes of physical activity/week (MD: 59.96; 95% CI 15.67, 104.25) associated with interventions. There was some evidence for beneficial effects on peak VO2, blood pressure, total cholesterol and mental health. Variation in outcome measurements reported for other behavioural risk factors limited the ability to perform meta-analyses. Effective interventions were based in homes, general practices or outpatient settings, individually tailored and often multicomponent with a theoretical framework. The review identified evidence that interventions for secondary CVD prevention, delivered in various community-based venues, have positive effects on physical activity; such opportunities should be promoted by health professionals. |
| Simpson V., Pedigo L. Nurse and Physician Involvement in Health Risk Appraisals: An Integrative Review. Western Journal of Nursing Research. 2017;39(6):803-824. doi:10.1177/0193945916660341 | To examine nurse and physician use of health risk appraisals in primary care. | A total of 26 national and international papers, selected through an electronic database and ancestry search, were reviewed. Identified nurse and physician interventions in addition to other programming included helping participants understand and interpret feedback, behavioral counseling, and development of plans to address unhealthy lifestyle behaviors. The most common intervention was provision of telephonic nurse advice lines. Overall outcomes were positive. The use of these tools could be key to enhancing primary care prevention. |
| Moran P. S., Teljeur C., Ryan M., Smith S. M. Systematic screening for the detection of atrial fibrillation. Cochrane Database of Systematic Reviews 2016, Issue 6. Art. No.: CD009586. DOI: 10.1002/14651858.CD009586.pub3. Accessed 17 May 2022. | To answer the following questions.  Does systematic screening increase the detection of AF compared with routine practice? Which combination of screening population, strategy and test is most effective for detecting AF compared with routine practice? What safety issues and adverse events may be associated with individual screening programmes? How acceptable is the intervention to the target population? What costs are associated with systematic screening for AF? | One cluster‐randomised controlled trial met the inclusion criteria for this review. This study compared systematic screening (by invitation to have an electrocardiogram (ECG)) and opportunistic screening (pulse palpation during a general practitioner (GP) consultation for any reason, followed by an ECG if pulse was irregular) versus routine practice (normal case finding on the basis of clinical presentation) in people 65 years of age or older. Results show that both systematic screening and opportunistic screening of people over 65 years of age are more effective than routine practice (OR 1.57, 95% CI 1.08 to 2.26; and OR 1.58, 95% CI 1.10 to 2.29, respectively; both moderate‐quality evidence). The study found no difference in the effectiveness of systematic screening and opportunistic screening (OR 0.99, 95% CI 0.72 to 1.37; low‐quality evidence). A subgroup analysis found that systematic screening and opportunistic screening were more effective in men (OR 2.68, 95% CI 1.51 to 4.76; and OR 2.33, 95% CI 1.29 to 4.19, respectively) than in women (OR 0.98, 95% CI 0.59 to 1.62; and OR 1.2, 95% CI 0.74 to 1.93, respectively). No adverse events associated with screening were reported. The incremental cost per additional case detected by opportunistic screening was GBP 337, compared with GBP 1514 for systematic screening. All cost estimates were based on data from the single included trial, which was conducted in the UK between 2001 and 2003. |
| Martin A., Saunders C. L., Harte E., Griffin S. J., MacLure C., Mant J., Meads C., Walter F. M., Usher-Smith J. A. Delivery and impact of the NHS Health Check in the first 8 years: a systematic review. Br J Gen Pract. 2018 Jul;68(672):e449-e459. doi: 10.3399/bjgp18X697649. Epub 2018 Jun 18. PMID: 29914882; PMCID: PMC6014431 | To review quantitative evidence on coverage (the proportion of eligible individuals who attend), uptake (proportion of invitees who attend), and impact of NHS Health Checks. | Twenty-six observational studies and one additional dataset were included. Since 2013, 45.6% of eligible individuals have received a health check. Coverage is higher among older people, those with a family history of coronary heart disease, those living in the most deprived areas, and some ethnic minority groups. Just under half (48.2%) of those invited have taken up the invitation. Data on uptake and impact (especially regarding health-related behaviours) are limited. Uptake is higher in older people and females, but lower in those living in the most deprived areas. Attendance is associated with small increases in disease detection, decreases in modelled cardiovascular disease risk, and increased statin and antihypertensive prescribing |
| Engelsen Cd., Koekkoek P. S., Godefrooij M. B., Spigt M. G., Rutten G.E. Screening for increased cardiometabolic risk in primary care: a systematic review. Br J Gen Pract. 2014 Oct;64(627):e616-26. doi: 10.3399/bjgp14X681781. PMID: 25267047; PMCID: PMC4173724. | To present a systematic review of cardiometabolic screening programmes performed among apparently healthy people (not yet known to have CVD, diabetes, or cardiometabolic risk factors) and mixed populations (apparently healthy people and people diagnosed with risk factor or disease) to define the optimal screening strategy. | The search strategy yielded 11 445 hits; 26 met the inclusion criteria. Five studies (1995–2012) were conducted in apparently healthy populations: three used a stepwise method. Response rates varied from 24% to 79%. Twenty-one studies (1967–2012) were performed in mixed populations; one used a stepwise method. Response rates varied from 50% to 75%. Prevalence rates could not be compared because of heterogeneity of used thresholds and eligible populations. Observed time trends were a shift from mixed to apparently healthy populations, increasing use of risk scores, and increasing use of stepwise screening methods. |
| . Rodrigues A. L., Ball J., Ski C., Stewart S., Carrington M. J. A systematic review and metaanalysis of primary prevention programmes to improve cardio-metabolic risk in non-urban communities. Prev Med. 2016 Jun;87:22-34. doi: 10.1016/j.ypmed.2016.02.011. Epub 2016 Feb 12. PMID: 26876624. | To review the effectiveness of primary prevention programmes aimed at reducing risk factors for CVD/T2DM, including blood pressure, body mass index (BMI), blood lipid and glucose, diet, lifestyle, and knowledge in adults residing in non-urban areas | Multiple strategies within interventions focusing on health behaviour change effectively reduced cardio-metabolic risk in non-urban individuals. Pre-/post-test design studies showed more favourable improvements generally, while RCTs showed greater improvements in physical activity and disease and risk knowledge. Short-term programmes were more effective than long-term programmes and in pre-/post-test designs reduced systolic blood pressure by 4.02 mm Hg (95% CI −6.25 to −1.79) versus 3.63 mm Hg (95% CI −7.34 to 0.08) in long-term programmes. Community-based programmes achieved good results for most risk factors except BMI and (glycated haemoglobin) HbA1c. |
| Lee J. T., Lawson K. D., Wan Y., Majeed A., Morris S., Soljak M., Millett C. Are cardiovascular disease risk assessment and management programmes cost effective? A systematic review of the evidence. Prev Med. 2017 Jun;99:49-57. doi: 10.1016/j.ypmed.2017.01.005. Epub 2017 Jan 11. PMID: 28087465. | The World Health Organization recommends that countries implement population-wide cardiovascular disease (CVD) risk assessment and management programmes. The aim of this study was to conduct a systematic review to evaluate whether this recommendation is supported by cost-effectiveness evidence | Programmes were either not, or only, cost-effective under non-verified assumptions such as sustained risk factor changes. Most observational and hypothetical studies suggested programmes were likely to be cost-effective; however, study deigns are subject to bias and subsequent empirical evidence has contradicted key assumptions. No studies assessed impacts on inequalities. In conclusion, recommendations for population-wide risk assessment and management programmes lack a robust, real world, evidence basis |
| Sheridan, S.L., Crespo, E. Does the routine use of global coronary heart disease risk scores translate into clinical benefits or harms? A systematic review of the literature. BMC Health Serv Res 8, 60 (2008). https://doi.org/10.1186/1472-6963-8-60 | Guidelines now recommend routine assessment of global coronary heart disease (CHD) risk scores. This systematic review assesses whether global CHD risk scores result in clinical benefits or harms | 11 studies met criteria for inclusion in the review. Six studies addressed clinical benefits and 5 addressed clinical harms. Six studies were rated as "fair" quality and the others were deemed "methodologically limited". Two fair quality studies showed that physician knowledge of global CHD risk is associated with increased prescription of cardiovascular drugs in high risk (but not all) patients. Two additional fair quality studies showed no effect on their primary outcomes, but one was underpowered and the other focused on prescribing of lifestyle changes, rather than drugs whose prescribing might be expected to be targeted by risk level. One of these aforementioned studies showed improved blood pressure in high-risk patients, but no improvement in the proportion of patients at high risk, perhaps due to the high proportion of participants with baseline risks significantly exceeding the risk threshold. Two fair quality studies found no evidence of harm from patient knowledge of global risk scores when they were accompanied by counseling, and optional or scheduled follow-up. Other studies were too methodologically limited to draw conclusions. |
| Goris J., Komaric N., Guandalini A., Francis D., Hawes E. Effectiveness of multicultural health workers in chronic disease prevention and self-management in culturally and linguistically diverse populations: a systematic literature review. Aust J Prim Health. 2013;19(1):14-37. doi: 10.1071/PY11130. PMID: 22951183. | The objective of this systematic review was to examine the effectiveness of multicultural health workers (MHWs) interventions in chronic disease prevention and self-management in culturally and linguistically diverse (CALD) populations with the aim to inform policy development of effective health care in CALD communities in Australia. | Thirty-nine studies were identified including 31 randomised controlled trials. Many of the studies focussed on poor and underserved ethnic minorities. Several studies reported significant improvements in participants' chronic disease prevention and self-management outcomes and meta-analyses identified a positive trend associated with MHW intervention. Australian Government policies express the need for targeted inventions for CALD communities. The broader systemic application of MHWs in Australian primary health care may provide one of the most useful targeted interventions for CALD communities. |
| Galaviz K. I., Weber M. B., Straus A., Haw J. S., Narayan K. M. V., Ali M. K. Global Diabetes Prevention Interventions: A Systematic Review and Network Meta-analysis of the RealWorld Impact on Incidence, Weight, and Glucose. Diabetes Care. 2018 Jul;41(7):1526- 1534. doi: 10.2337/dc17-2222. PMID: 29934481; PMCID: PMC6463613. | The purpose of this study was to synthetize global evidence on the impact of lifestyle modification strategies on diabetes incidence and risk factors in one parsimonious model. | Sixty-three studies were pooled in the meta-analysis (n = 17,272, mean age 49.7 years, 28.8% male, 60.8% white/European). In analyses restricted to controlled studies (n = 7), diabetes cumulative incidence was 9% among intervention participants and 12% among control participants (absolute risk reduction 3%; relative risk 0.71 [95% CI 0.58, 0.88]). In analyses combining controlled and uncontrolled studies (n = 14), participants receiving group education by health care professionals had 33% lower diabetes odds than control participants (odds ratio 0.67 [0.49, 0.92]). Intervention participants lost 1.5 kg more weight [-2.2, -0.8] and achieved a 0.09 mmol/L greater FBG decrease [-0.15, -0.03] than control participants. Every additional kilogram lost by participants was associated with 43% lower diabetes odds (β = 0.57 [0.41, 0.78]). |
| Bunten, A., Porter, L., Gold, N., Bogle, V. A systematic review of factors influencing NHS health check uptake: invitation methods, patient characteristics, and the impact of interventions. BMC Public Health 20, 93 (2020). https://doi.org/10.1186/s12889-019-7889-4 | The aim of this systematic review is to highlight interventions and invitation methods that increase the uptake of NHS Health Checks, and to identify whether the effectiveness of these interact with broader patient and contextual factors. | The nine studies were all from peer reviewed journals. They included two randomised controlled trials, one observational cohort and six cross-sectional studies. Different invitation methods may be more effective for different groups of patients based on their ethnicity and gender. One intervention to enhance invitation letters effectively increased uptake but another did not. In addition, individual patient characteristics (such as age, gender, ethnicity and risk level) were found to influence uptake. This review also finds that uptake varies significantly by GP practice, which could be due either to unidentified practice-level factors or deprivation. |
| Murray, J., Craigs, C.L., Hill, K.M., Honey, S., House, A. A systematic review of patient reported factors associated with uptake and completion of cardiovascular lifestyle behaviour change. BMC Cardiovasc Disord 12, 120 (2012). https://doi.org/10.1186/1471-2261-12-120 | The aim of the study was to clarify which influences reported by patients predict uptake and completion of formal lifestyle change programmes. | 374 factors were extracted from 32 studies. Factors most consistently associated with uptake of lifestyle change related to support from family and friends, transport and other costs, and beliefs about the causes of illness and lifestyle change. Depression and anxiety also appear to influence uptake as well as completion. Many factors show inconsistent patterns with respect to uptake and completion of lifestyle change programmes. |
| Schumacher T. L., Burrows T. L., Neubeck L., Redfern J., Callister R., Collins C. E. How dietary evidence for the prevention and treatment of CVD is translated into practice in those with or at high risk of CVD: a systematic review. Public Health Nutr. 2017 Jan;20(1):30-45. doi: 10.1017/S1368980016001543. Epub 2016 Jun 22. PMID: 27330027. | The aim of the present systematic review was to synthesise the literature relating to knowledge translation (KT) of dietary evidence for the prevention and treatment of CVD into practice in populations with or at high risk of CVD. | A total of 4420 titles and abstracts were screened for inclusion, with 354 full texts retrieved to assess inclusion. Forty-three articles were included in the review, relating to thirty-five separate studies. No studies specifically stated their aim to be KT. Thirty-one studies were in patient or high-risk populations and four targeted health professionals. Few studies stated a theory on which the intervention was based (n 10) and provision of instruction was the most common behaviour change strategy used (n 26). |
| Álvarez-Bueno C., Cavero-Redondo I., Martínez-Andrés M., Arias-Palencia N., RamosBlanes R., Salcedo-Aguilar F. Effectiveness of multifactorial interventions in primary health care settings for primary prevention of cardiovascular disease: A systematic review of systematic reviews. Prev Med. 2015 Jul;76 Suppl:S68-75. doi: 10.1016/j.ypmed.2014.11.028. 84 Epub 2014 Dec 12. PMID: 25511466 | To evaluate the effectiveness of multifactorial interventions carried out in the community setting to decrease cardiovascular risk in healthy patients. | Eight systematic reviews were selected, including 219 studies. All of these reviews provided information about the effectiveness of multifactorial interventions in reducing mortality and morbidity due to cardiovascular diseases. Four reviews reported moderate effectiveness and four showed limited effectiveness. |
| . Curtis J., Wilson C. Preventing type 2 diabetes mellitus. The Journal of the American Board of Family Practice. 2005. DOI:10.3122/JABFM.18.1.37 | To examine the evidence for different strategies aimed at preventing type 2 diabetes in patients with impaired glucose tolerance, impaired fasting glucose, obesity, gestational diabetes, hypertension, hyperlipidemia, and menopause. | The strongest evidence supports an intensive lifestyle intervention designed to induce modest weight loss. The greatest degree of prevention, based on lesser quality evidence, may be imparted by bariatric surgery. Metformin and troglitazone have appreciable evidence in specific populations, and orlistat and acarbose have slightly less evidence among obese patients, for preventing diabetes. Ramipril, captopril, losartan, pravastatin, and estrogens show some very preliminary promise for preventing diabetes in patients treated for hypertension, hyperlipidemia, and menopause, but each needs a more rigorous evaluation. Although more questions remain to be answered, family physicians now have tools available to help patients lead lives free of diabetes. |
| Willis A., Davies M., Yates T., Khunti K. Primary prevention of cardiovascular disease using validated risk scores: a systematic review. J R Soc Med. 2012 Aug;105(8):348-56. doi: 10.1258/jrsm.2012.110193. Erratum in: J R Soc Med. 2012 Oct;105(10):411. PMID: 22907552; PMCID: PMC3423129. | This review summarizes current evidence for and against the use of validated CVD risk scores for the primary prevention of CVD. | The study identified 16 papers which matched the inclusion criteria reporting 5 unique trials. Due to a lack of homogeneity in outcomes and risk scores used it was not possible to conduct a meta-analysis of the identified studies. Only one study reported a significant difference in risk score at follow up and one study reported a significant difference in total mortality, however significant differences in individual risk factors were reported by the majority of identified studies. |
| Waugh N. R., Shyangdan D., Taylor-Phillips S., Suri G., Hall B. Screening for type 2 diabetes: a short report for the National Screening Committee. Health Technol Assess. 2013 Aug;17(35):1-90. doi: 10.3310/hta17350. PMID: 23972041; PMCID: PMC4780946. | The aim of this review was to provide an update for the UK National Screening Committee (NSC) on screening for T2DM. | Population screening for T2DM does not meet all of the NSC criteria. Criterion 12, on optimisation of existing management, has not been met. A report by the National Audit Office (NAO) gives details of shortcomings. Criterion 13 requires evidence from high-quality randomised controlled trials that screening is beneficial. This has not been met. The Ely trial of screening showed no benefit. The ADDITION trial was not a trial of screening, but showed no benefit in cardiovascular outcomes from intensive management in people with screen-detected T2DM. Criterion 18 on staffing and facilities does not appear to have been met, according to the NAO report. Criterion 19 requires that all other options, including prevention, should have been considered. A large proportion of cases of T2DM could be prevented if people avoided becoming overweight or obese. The first stage of selection would use risk factors, using data held on general practitioner computer systems, using the QDiabetes Risk Score, or by sending out questionnaires, using the Finnish Diabetes Risk Score (FINDRISC). Those at high risk would have a measure of blood glucose. There is no perfect screening test. Glycated haemoglobin (HbA1c) testing has advantages in not requiring a fasting sample, and because it is a predictor of vascular disease across a wider range than just the diabetic one. However, it lacks sensitivity and would miss some people with diabetes. Absolute values of HbA1c may be more useful as part of overall risk assessment than a dichotomous 'diabetes or not diabetes' diagnosis. The oral glucose tolerance test is more sensitive, but inconvenient, more costly, has imperfect reproducibility and is less popular, meaning that uptake would be lower. |
| Dyakova M., Shantikumar S., Colquitt J. L., Drew C. M., Sime M., MacIver J., Wright N., Clarke A., Rees K. Systematic versus opportunistic risk assessment for the primary prevention of cardiovascular disease. Cochrane Database Syst Rev. 2016 Jan 29;2016(1):CD010411. doi: 10.1002/14651858.CD010411.pub2. PMID: 26824223; PMCID: PMC6494380. | The primary objective of this review was to assess the effectiveness, costs and adverse effects of systematic risk assessment compared to opportunistic risk assessment for the primary prevention of CVD. | Nine completed RCTs met the inclusion criteria, of which four were cluster-randomised. The study also identified five ongoing trials. The included studies had a high or unclear risk of bias, and the GRADE ratings of overall quality were low or very low. The length of follow-up varied from one year in four studies, three years in one study, five or six years in two studies, and ten years in two studies. Eight studies recruited participants from the general population, although there were differences in the age ranges targeted. One study recruited family members of cardiac patients (high risk assessment). There were considerable differences between the studies in the interventions received by the intervention and control groups. There was insufficient evidence to stratify by the types of risk assessment approaches.Limited data were available on all-cause mortality (risk ratio (RR) 0.97, 95% confidence interval (CI) 0.92 to 1.02; 3 studies,103,571 participants, I² = 0%; low-quality evidence) and cardiovascular mortality (RR 1.00, 95% CI 0.90 to 1.11; 2 studies, 43,955 participants, I² = 0%), and suggest that screening has no effect on these outcomes. Data were also limited for combined non-fatal endpoints; overall, evidence indicates no difference in total coronary heart disease (RR 1.01, 95% CI 0.95 to 1.07; 4 studies, 5 comparisons, 110,168 participants, I² = 0%; low-quality evidence), non-fatal coronary heart disease (RR 0.98, 95% CI 0.89 to 1.09; 2 studies, 43,955 participants, I² = 39%), total stroke (RR 0.99, 95% CI 0.90 to 1.10; 2 studies, 79,631 participants, I² = 0%, low-quality evidence), and non-fatal stroke (RR 1.17, 95% CI 0.94 to 1.47; 1 study, 20,015 participants).Overall, systematic risk assessment appears to result in lower total cholesterol levels (mean difference (MD) -0.11 mmol/l, 95% CI -0.17 to -0.04, 6 studies, 7 comparisons, 12,591 participants, I² = 57%; very low-quality evidence), lower systolic blood pressure (MD -3.05 mmHg, 95% CI -4.84 to -1.25, 6 studies, 7 comparisons, 12,591 participants, I² = 82%; very low-quality evidence) and lower diastolic blood pressure (MD -1.34 mmHg, 95% CI -1.76 to -0.93, 6 studies, 7 comparisons, 12,591 participants, I² = 0%; low-quality evidence). One study assessed adverse effects and found no difference in psychological distress at five years (1126 participants). |
| Crouch R., Wilson A., Newbury J. A systematic review of the effectiveness of primary health education or intervention programs in improving rural women's knowledge of heart disease risk factors and changing lifestyle behaviours. Int J Evid Based Healthc. 2011 Sep;9(3):236-45. doi: 10.1111/j.1744-1609.2011.00226.x. PMID: 21884451. | To determine the effectiveness of primary health education or intervention programs for cardiac risk reduction in healthy women living in rural areas. | Nine trials were included in the review. Three trials compared the effects of interventions on physical activity, one on smoking and five on multiple risk factors. Studies following interventions targeting physical activity reported that women's physical activity can be increased and that these increases can be sustained at 12 months. While there were decreases in blood pressure at 6 months, studies with a 5-year follow up found no decreases for both systolic and diastolic blood pressure. Overall results of studies into dietary modification programs also did not sustain an effect over a longer period of time. |
| Buckley B. S., Byrne M. C., Smith S. M. Service organisation for the secondary prevention of ischaemic heart disease in primary care. Cochrane Database Syst Rev. 2010 Mar 17;(3):CD006772. doi: 10.1002/14651858.CD006772.pub2. PMID: 20238349. | To assess the effectiveness of service organisation interventions, identifying which types and elements of service change are associated with most improvement in clinician and patient adherence to secondary prevention recommendations relating to risk factor levels and monitoring (blood pressure, cholesterol and lifestyle factors such as diet, exercise, smoking and obesity) and appropriate prophylactic medication. | Eleven studies involving 12,074 people with IHD were included. Increased proportions of patients with total cholesterol levels within recommended levels at 12 months, OR 1.90 (1.04 to 3.48), were associated with interventions that included regular planned appointments, patient education and structured monitoring of medication and risk factors, but significant heterogeneity was apparent. Results relating to blood pressure within target levels bordered on statistical significance. There were no significant effects of interventions on mean blood pressure or cholesterol levels, prescribing, smoking status or body mass index. Few data were available on the effect on diet. There was some suggestion of a "ceiling effect" whereby interventions have a diminishing beneficial effect once certain levels of risk factor management are reached. |
| Wändell P. E., de Waard A. K. M., Holzmann M. J., Gornitzki C., Lionis C., de Wit N., Søndergaard J., Sønderlund A. L., Kral N., Seifert B., Korevaar J. C., Schellevis F. G., Carlsson A. C., Barriers and facilitators among health professionals in primary care to prevention of cardiometabolic diseases: A systematic review, Family Practice, Volume 35, Issue 4, August 2018, Pages 383-398, https://doi.org/10.1093/fampra/cmx137 | The aim of this study is to identify potential facilitators and barriers for health care professionals to undertake selective prevention of cardiometabolic diseases (CMD) in primary health care. | The study found 19 qualitative studies, 7 quantitative studies and 2 mixed qualitative and quantitative studies. In terms of five overarching categories, the most frequently reported barriers and facilitators were as follows: Structural (barriers: time restraints, ineffective counselling and interventions, insufficient reimbursement and problems with guidelines; facilitators: feasible and effective counselling and interventions, sufficient assistance and support, adequate referral, and identification of obstacles), Organizational (barriers: general organizational problems, role of practice, insufficient IT support, communication problems within health teams and lack of support services, role of staff, lack of suitable appointment times; facilitators: structured practice, IT support, flexibility of counselling, sufficient logistic/practical support and cooperation with allied health staff/community resources, responsibility to offer and importance of prevention), Professional (barriers: insufficient counselling skills, lack of knowledge and of experience; facilitators: sufficient training, effective in motivating patients), Patient-related factors (barriers: low adherence, causes problems for patients; facilitators: strong GP-patient relationship, appreciation from patients), and Attitudinal (barriers: negative attitudes to prevention; facilitators: positive attitudes of importance of prevention) |
| Mills, K., Harte, E., Martin, A., MacLure, C., Griffin, S. J., Mant, J., Meads, C., Saunders, C. L., Walter, F. M., & Usher-Smith, J. A., Views of commissioners, managers and healthcare professionals on the NHS Health Check programme: a systematic review. 2017, BMJ open, 7(11), e018606. https://doi.org/10.1136/bmjopen-2017-018606 | To synthesise data concerning the views of commissioners, managers and healthcare professionals towards the National Health Service (NHS) Health Check programme in general and the challenges faced when implementing it in practice. | Of 18 524 citations, 15 articles met the inclusion criteria. There was evidence from both quantitative and qualitative studies that some commissioners and general practice (GP) healthcare professionals were enthusiastic about the programme, whereas others raised concerns around inequality of uptake, the evidence base and cost-effectiveness. In contrast, those working in pharmacies were all positive about programme benefits, citing opportunities for their business and staff. The main challenges to implementation were: difficulties with information technology and computer software, resistance to the programme from some GPs, the impact on workload and staffing, funding and training needs. Inadequate privacy was also a challenge in pharmacy and community settings, along with difficulty recruiting people eligible for Health Checks and poor public access to some venues. |
| Si S., Moss J. R., Sullivan T. R., Newton S. S., Stocks N. P. Effectiveness of general practice-based health checks: a systematic review and meta-analysis. Br J Gen Pract. 2014 Jan;64(618):e47-53. doi: 10.3399/bjgp14X676456. PMID: 24567582; PMCID: PMC3876170. | This review focuses on general practice-based health checks and their effects on both surrogate and final outcomes. | Six trials were included. The end-point differences between the intervention and control arms in total cholesterol (TC), systolic and diastolic blood pressure (SBP, DBP), and body mass index (BMI) were -0.13 mmol/l (95% confidence interval [CI] = -0.19 to -0.07), -3.65 mmHg (95% CI = -6.50 to -0.81), -1.79 mmHg (95% CI = -2.93 to -0.64), and -0.45 kg/m(2) (95% CI = -0.66 to -0.24), respectively. The odds of a patient remaining at 'high risk' with elevated TC, SBP, DBP, BMI or continuing smoking were 0.63 (95% CI = 0.50 to 0.79), 0.59 (95% CI = 0.28 to 1.23), 0.63 (95% CI = 0.53 to 0.74), 0.89 (95% CI = 0.81 to 0.98), and 0.91 (95% CI = 0.82 to 1.02), respectively. There was little evidence of a difference in total mortality (OR 1.03, 95% CI = 0.90 to 1.18). Higher CVD mortality was observed in the intervention group (OR 1.30, 95% CI = 1.02 to 1.66). |
| Wolfenden, L.; Reilly, K.; Kingsland, M.; Grady, A.; Williams, C. M.; Nathan, N.; Sutherland, R.; Wiggers, J.; Jones, J.; Hodder, R.; Finch, M.; McFadyen, T.; Bauman, A.; Rissel, C.; Milat, A.; Swindle, T.; Yoong SzeLin. Identifying opportunities to develop the science of implementation for community-based non-communicable disease prevention: a review of implementation trials. 2019, Preventive Medicine 118, 279–285, https://doi.org/10.1016/j.ypmed.2018.11.014 | This study aimed to characterise experimental research regarding strategies to improve implementation of chronic disease prevention programs in community settings. | Of the 40 implementation trials included in the study, unhealthy diet was the most common risk factor targeted (n=20). The most commonly reported implementation strategies were educational meetings (n=38, 95%), educational materials (n=36, 90%) and educational outreach visits (n=29, 73%). Few trials were conducted 'at-scale' (n=8, 20%) or reported adverse effects (n=5, 13%). The reporting of implementation related outcomes; intervention adoption (n=13, 33%); appropriateness (n=11, 28%); acceptability (n=8, 20%); feasibility (n=8, 20%); cost (n=3, 8%); and sustainability (n=2, 5%); was limited. For the majority of trials, risk of bias was high for blinding of study personnel/participants and outcome assessors |
| Koopmans, B.; Nielen, M. M. J.; Schellevis, F. G.; Korevaar, J. C. Non-participation in population-based disease prevention programs in general practice. 2012, BMC public health. 12. 856. 10.1186/1471-2458-12-856. | The aim of this systematic review was to identify factors that negatively influence participation in population-based disease prevention programs in General Practice and to establish whether the program type is related to non-participation levels. | A total of 24 original studies met the criteria, seven of which focused on vaccination, eleven on screening aimed at early detection of disease, and six on screening aimed at identifying high risk of a disease, targeting a variety of diseases and conditions. Lack of personal relevance of the program, younger age, higher social deprivation and former non-participation were related to actual non-participation. No ifferences were found in non-participation levels or factors related to non-participation between the three program types. The large variation in non-participation levels within the program types may be partly due to differences in recruitment strategies, with more active, personalized trategies resulting in higher participation levels compared to an invitation letter. |
| Goldfarb M., Slobod D., Dufresne L., Brophy J. M., Sniderman A., Thanassoulis G. Screening Strategies and Primary Prevention Interventions in Relatives of People With Coronary Artery Disease: A Systematic Review and Meta-analysis. Can J Cardiol. 2015 May;31(5):649-57. doi: 10.1016/j.cjca.2015.02.019. Epub 2015 Feb 20. PMID: 25936490. | To examine screening strategies targeting relatives of people with coronary artery disease | The study identified 18 studies that reported screening strategies and 15 reporting interventions to reduce CV risk. Proband willingness to refer relatives for screening was high (n = 6 studies, pooled rate = 87%; 95% confidence interval [CI], 80%-95%). Studies using a screening strategy in which the relative was contacted by health care professionals reported a pooled participation rate of 88% (95% CI, 78%-99%). The quality of interventional studies was highly variable. Random-effects meta-analysis of the highest quality randomized studies (n = 6) consisting of a specialized risk factor intervention compared with usual care was consistent with modest improvements in low-density lipoprotein cholesterol control (-0.18 mmol/L low-density lipoprotein cholesterol, 95% CI, -0.35 to -0.001; P = 0.048). Improvements in diet, smoking rates, exercise, and blood pressure were also observed with active intervention; however, reported outcomes were heterogeneous precluding a formal meta-analysis. |
| Sisti, L. G., Dajko, M., Campanella, P., Shkurti, E., Ricciardi, W., Waure, C. de. The effect of multifactorial lifestyle interventions on cardiovascular risk factors: a systematic review and meta-analysis of trials conducted in the general population and high risk groups. Preventive Medicine. 109. 10.1016/j.ypmed.2017.12.027. | To evaluate the impact of multifactorial lifestyle interventions on cardiovascular risk modification, both in the general and risk population. | Search resulted in 19,847 studies, of which 36 were included in the analysis. Compared to a usual care, the multifactorial lifestyle intervention is able to lower the blood pressure, total cholesterol, BMI and waist circumference, at both 6 and 12months, and to increase physical activity at 12months. Better results were obtained in primary prevention and in moderate and high risk groups. |
| Jepson, R.G., Harris, F.M., Platt, S., Tannahill, C. The effectiveness of interventions to change six health behaviours: a review of reviews. BMC Public Health 10, 538 (2010). https://doi.org/10.1186/1471-2458-10-538 | To review reviews of behavioural change interventions to reduce unhealthy behaviours or promote healthy behaviours. | The study included 103 reviews published between 1995 and 2008. The focus of interventions varied, but those targeting specific individuals were generally designed to change an existing behaviour (e.g. cigarette smoking, alcohol misuse), whilst those aimed at the general population or groups such as school children were designed to promote positive behaviours (e.g. healthy eating). Almost 50% (n = 48) of the reviews focussed on smoking (either prevention or cessation). Interventions that were most effective across a range of health behaviours included physician advice or individual counselling, and workplace- and school-based activities. Mass media campaigns and legislative interventions also showed small to moderate effects in changing health behaviours.  Generally, the evidence related to short-term effects rather than sustained/longer-term impact and there was a relative lack of evidence on how best to address inequalities. |
| de Waard A. K. M., Wändell, P. E., Holzmann, M. J., Korevaar, J. C., Hollander, M., Gornitzki, C., de Wit, N. J., Schellevis, F. G., Lionis, C., Søndergaard, J., Seifert, B., Carlsson, A. C. on behalf of the SPIMEU Research Group, Barriers and facilitators to participation in a health check for cardiometabolic diseases in primary care: A systematic review, European Journal of Preventive Cardiology, Volume 25, Issue 12, 1 August 2018, Pages 1326–1340, | To examine barriers and facilitators determining participation in health checks in primary care. | Thirty-nine studies were included. Attitudes such as wanting to know of cardiometabolic disease risk, feeling responsible for, and concerns about one’s own health were facilitators for participation. Younger age, smoking, low education and attitudes such as not wanting to be, or being, worried about the outcome, low perceived severity or susceptibility, and negative attitude towards health checks or prevention in general were barriers. Furthermore, practical issues such as information and the ease of access to appointments could influence participation. |

| **Phase 3: Literature review of qualitative studies and grey literature (grey literature)** | | | | |
| --- | --- | --- | --- | --- |
| Author/year | Country | Aim | Intervention characteristics | Main results |
| Bjerregaard, A. L., Dalsgaard, E., Norman, K., Larsen, L. B., Merrild C.H. 2020. “Dit liv – din sundhed.” Aarhus: Institut for Folkesundhed, Aarhus Universitet. | Denmark | To describe the research project “Dit Liv – Din Sundhed” [“Your Life – Your Health”]. | Health checks and subsequent behaviour-change interventions, and/or medical treatment targeting residents from the Danish social housing sector. The health checks took place in municipal health centres. | No results as the aim was to describe the intervention. |
| Bjerregaard, A. L., and K. Norman. 2017. "Sund Mand - Evalueringsrapport 2017." Aarhus: Institut for Folkesundhed, Aarhus Universitet. | Denmark | To 1) describe the sociodemographic and health-related characteristics of the participants in the intervention “Sund Mand”[“Healthy Man”], and 2) explore whether the intervention has motivated the participants to change their health-related behaviour regarding physical activity, smoking and alkohol intake | In a Danish municipality, men at age 40, 45 and 50 were invited for a health check focusing on early detection of cardiovascular disease and mental health problems. The health checks were carried out at a municipal health center, and participants at risk were referred to a follow-up consultation in the municipal health center or with their general practitioner. | 2/3 of the participants were married, 62 % had a short education, 1/5 lived alone. 74 % had a risk profile requiring lifestyle changes and/or medical treatment. One year after the intervention, the participants filled out a survey. The survey showed improvements regarding smoking habits, physical activity and alcohol intake. A majority of the participants state that the intervention had a positive impact on them. |
| Bjerregaard, A. L., and K. Norman. 2017. Tjek dit helbred – Evalueringsrapport 2017. Aarhus Universitet. | Denmark | To 1) describe the health profile of participants in “Tjek dit helbred” [“Check your health”] within the first four years, 2) describe the pattern of attendance and non-attendance in health check based on information from national databases and survey data, 3) discuss design of future health check interventions, including which possible target group to approach and which components should be incorporated in future interventions | “Tjek dit helbred” [“Check your health”] offered preventive health check and health consultations to citizens aged 30-49 years old in a Danish municipality. The aim of the health check was to detect risk of cardiovascular diseases and mental health problems. Participants at risk were offered a follow-up consultation either with their general practitioner or in a municipal setting. | Factors influencing uptake were income, education, and whether you live alone. 85 % had a risk profile requiring lifestyle changes and/or medical treatment. |
| Broholm-Jørgensen, M., Kamstrup-Larsen, N., 2020. “Tjek ind – forebyggende helbredstjek i almen praksis.” Center for Interventionsforskning,Statens Institut for Folkesundhed, SDU. | Denmark | To provide an overview of the research project “Tjek Ind”[“Check In”] including main results. | Individuals with no formal education beyond lower secondary school and aged 45–64 years were invited to a prescheduled preventive health check from the general practitioner (GP) followed by a health consultation and an offer of a follow-up with health risk behaviour change or preventive medical treatment, if necessary. | Overall, the health check intervention had no effect on smoking habits, alcohol intake, physical activity and obesity. The detection of depression, however, was three times more likely in the intervention group than in the control group.  Daily smoking, poor self-reported health status, high level of stress and lack of contact with general practice were related to non-attendance in the intervention.  Qualitative insights from the research project shows that health consultations do not necessarily foster a change in health behaviours. |
| KORA. 2014. "En omkostningsanalyse af den indledende fase af ’Tjek dit helbred’ i Randers Kommune." | Denmark | To 1) estimate the costs of implementing the intervention “Tjek dit helbred”[“Check Your Health”] and 2) compare the public expenses of the intervention group compared with the expenses of the control group | “Tjek dit helbred” [“Check your health”] offered preventive health check and health consultations to citizens aged 30-49 years old in a Danish municipality. The aim of the health check was to detect risk of cardiovascular diseases and mental health problems. Participants at risk were offered a follow-up consultation either with their general practitioner or in a municipal setting. | There is no significant difference between the public expenses of the intervention group and the control group. |
| Mølbak, M. L., Juhl, S., Abrahamsen, K. L.. 2019. Midtvejsevaluering - “Sundhedstjek: styrket rekruttering til kommunale sundhedstilbud”, COWI og Sundhedsstyrelsen. | Denmark | Mid-term evaluation of 11 municipalities’ efforts to offer health checks and follow-up activities. | 11 municipalities in Denmark invited 40-60-year-old people for a preventive health check and follow-up activities in a municipal setting. | Organizational issues have challenged the implementation of the intervention in some municipalities. Moreover, a narrow and targeted recruitment strategy has proven to me more successful than a broad recruitment strategy. Furthermore, it improves the implementation when the municipalities create a close cooperation with partner organizations. The mid-term evaluation shows that the municipalities succeed in recruiting participants from the target group. |
| Rasmussen, S. R., J. Kilsmark, A. Hvenegaard, J. L. Thomsen, M. Engberg, T. Lauritzen, and J. Søgaard. 2006. "FOREBYGGENDE HELBREDSUNDERSØGELSER OG HELBREDSSAMTALER I ALMEN PRAKSIS - en sundhedsøkonomisk analyse af ”Sundhedsprojekt Ebeltoft”." Medicinsk Teknologivurdering - puljeprojekter 2006; 6 (6). | Denmark | To explore whether it would be cost effective to implement preventive health checks and health consultations in Denmark. This is explored through an examination of the intervention called “The Ebeltoft Health Promotion Project” | A random sample of 2000 people aged between 30 and 50 were asked to participate in a population-based randomized, controlled health-promotion study in Ebeltoft, Denmark. They were offered a multiphasic risk factor screening in general practice and written feedback. One intervention group had a health discussion planned a few weeks after the screening, whereas the other intervention group could make an appointment for a consultation with their GP if they wished to. | The health effect of the intervention group was statistically significant compared to the control group after 6 years. Moreover, the intervention group did not have statistically significantly higher public expenses than the control group after 6 years. |
| Sundhedsstyrrelsen og NIRAS. 2015. “Erfaringer med at løfte sundheden i nærmiljøet" | Denmark | A cross-cut evaluation of 12 prevention and health promotion projects in 12 different municipalities in Denmark | 12 municipalities in Denmark have carried out preventive and health promoting activities among people with low socioeconomic status. The activities vary. | All 12 municipalities have succeeded in improving the health-related behaviour of a number of citizens. These changes include mproved well-being, physical activity, smoking habits, and eating habits. |
| Sundhedsstyrelsen og Cowi. 2019. "Evaluering - Tidlig opsporing af sygdom hos borgere med betydelig kognitive og psykiske funktionsevnenedsættelser." | Denmark | An evaluation of four municipal projects aiming at early detection of illnesses among people with mental or cognitive disabilities. | Preventive health checks carried out in general practice among people with mental or cognitive disabilities. | All four municipalities have succeeded in recruiting people with mental or cognitive disabilities for health checks in general practice, where physical and mental health problems have been detected. However, the municipalities have had difficulties recruiting the target group. |
| Thilsing, T., Svensson, N. H., Søndergaard, J., Larsen, L. B. 2020. "Evalueringsrapport for TOF, pilotprojekt 2 - Resultater af den kvantitative evaluering." | Denmark | A quantiative evaluation of the intervention ‘TOF’ (a Danish acronym for ‘Early Detection and  Prevention’) pilot project 2. | A Danish primary preventive intervention ‘TOF’ (a Danish acronym for ‘Early Detection and  Prevention’) carried out in 2016. The intervention consisted of 1) a stratification of patients into one of four groups,  2) a digital support system for both general practitioners and patients, 3) an individual digital health profile for each  patient, and 4) targeted preventive services in either general practice or a municipal health center. | Results include: 66 % of the target group accepted the invitation. According to the general practitioner, 46 % of the participants needed a medical health assessment aiming at lifestyle changes. The municipal employees considered the phone call to be relevant in 85 % of the cases. Approximately 1 in 5 participants reported that they had obtained a healthier lifestyle during the intervention period. Among the participants labelled “red”, 1 in 3 reported that they had obtained a healthier lifestyle during the intervention period. |

| **Phase 3: Literature review of qualitative studies and grey literature (qualitative studies)** | | | | |
| --- | --- | --- | --- | --- |
| Author/year | Country | Aim | Intervention characteristics | Main results |
| Bach Nielsen, Karen-Dorthe, Lise Dyhr, Torsten Lauritzen, and Kirsti Malterud. 2005. "Long-term impact of elevated cardiovascular risk detected by screening." Scandinavian Journal of Primary Health Care 23 (4):233-238. doi: 10.1080/02813430500336245. | Denmark | To explore how persons with an elevated cardiovascular risk score (CRS) balanced health-related advice against the life they wanted to live or were able to live. | A random sample of 2000 people aged between 30 and 50 were asked to participate in a population-based randomized, controlled health-promotion study in Ebeltoft, Denmark. They were offered a multiphasic risk factor screening in general practice and written feedback. One intervention group had a health discussion planned a few weeks after the screening, whereas the other intervention group could make an appointment for a consultation with their GP if they wished to. | Being informed about an elevated CRS had a considerable impact on the informants. They initiated significant lifestyle changes though only to a limited degree when such changes would affect their quality of life adversely. In cases where other results of the multiphasic screening were normal, interpreted as such, or if there were stressful circumstances in the informant’s life, the elevated CRS receded into the background. |
| Brangan, Emer, Tracey J. Stone, Amanda Chappell, Vivienne Harrison, and Jeremy Horwood. 2019. "Patient experiences of telephone outreach to enhance uptake of NHS Health Checks in more deprived communities and minority ethnic groups: A qualitative interview study." Health Expectations: An International Journal of Public Participation in Health Care & Health Policy 22 (3):364-372 | UK | To explore the experiences of patients who received an outreach call. | NHS Health Check: Local authorities are responsible for offering an NHS Health Check every 5 years to individuals aged 40-74 who are not on a relevant disease register. The programme aims to prevent heart disease, stroke, type two diabetes and kidney disease, using a combination of risk assessment, communication of risk and risk management | The call increased participants’ understanding of NHS Health Checks and overcame anticipated difficulties with making an appointment. Half reported that they would not have booked if only invited by letter. The cultural identity/language skills of the caller were important in facilitating the interaction for some who might otherwise encounter language or cultural barriers. The inclusion of lifestyle questions and signposting prompted a minority to make lifestyle changes |
| Broholm-Jørgensen, M., S. M. Langkilde, T. Tjørnhøj-Thomsen, and P. V. Pedersen. 2020. "'Motivational work': A qualitative study of preventive health dialogues in general practice." BMC Family Practice 21 (1). doi: 10.1186/s12875-020-01249-z. | Denmark | The aim of the article is to provide in-depth insight into the unfolding of preventive health dialogues in general practice from perspectives of both GPs and patients | A pilot study of a Danish primary preventive intervention ‘TOF’ (a Danish acronym for ‘Early Detection and Prevention’) carried out in 2016. The intervention consisted of 1) a stratification of patients into one of four groups, 2) a digital support system for both general practitioners and patients, 3) an individual digital health profile for each patient, and 4) targeted preventive services in either general practice or a municipal health center. | While the health dialogues in TOF sought to reveal patients’ motivations, understandings, and priorities related to health behavior, the study finds that the dialogues were treatment-oriented and structured around biomedical facts, numeric standards, and risk factor guidance. Overall, the study finds that numeric standards and quantification of motivation lessens the dialogue and interaction between General Practitioner and patient and that contextual factors relating to the intervention framework, such as a digital support system, the general practitioners’ perceptions of their professional position as well as the patients’ understanding of prevention —in an interplay— diminished the motivational work carried out in the health dialogues. |
| Corrrigan, M., M. E. Cupples, S. M. Smith, M. Byrne, C. S. Leathem, P. Clerkin, and A. W. Murphy. 2006. "The contribution of qualitative research in designing a complex intervention for secondary prevention of coronary heart disease in two different healthcare systems." BMC Health Serv Res 6:90. doi: 10.1186/1472-6963-6-90. | Northern Ireland and the Republic of Ireland | To examine the contribution of qualitative research in developing a complex intervention to improve the provision and uptake of secondary prevention of CHD within primary care in two different healthcare systems | N/R | Integrating qualitative research into the development of the intervention provided depth of information about the varying impact, between the two healthcare systems, of different funding and administrative arrangements, on their provision of secondary prevention and identified similar barriers of time constraints, training needs and poor patient motivation. The findings also highlighted the importance to patients of stress management, the need for which had been underestimated by the researchers. The qualitative evaluation provided depth of detail not found in evaluation questionnaires. It highlighted how the intervention needed to be more practical by minimising administration, integrating role plays into behaviour change training, providing more practical information about stress management and removing self-monitoring of lifestyle change |
| Eastwood, Sophie V., Greta Rait, Mimi Bhattacharyya, Devaki R. Nair, and Kate Walters. 2013. "Cardiovascular risk assessment of South Asian populations in religious and community settings: A qualitative study." Family Practice 30 (4):466-472. | UK | To use stakeholders’ and attendees’ experiences to explore the feasibility and potential impact of cardiovascular risk assessment targeting South Asian groups at religious and community venues and how health checks in these settings might compare with general practice assessments. | NHS Health Check: A 40-minute appointment, including anthropometrics, blood pressure, point-of-care cholesterol and glucose testing and lifestyle assessment, followed by tailored advice on lifestyle improvement. | All attendees reported positive experiences of the assessments. All reported making lifestyle changes after the check, particularly to diet and exercise. Barriers to lifestyle change, e.g. resistance to change from family members, were identified. Advantages of implementing assessments in religious and community settings compared with general practice included accessibility and community encouragement. Disadvantages included reduced privacy, organizational difficulties and lack of follow-up care. |
| Eborall, Helen, Richard Davies, Ann-Louise Kinmonth, Simon Griffin, and Julia Lawton. 2007. "Patients' experiences of screening for type 2 diabetes: Prospective qualitative study embedded in the ADDITION (Cambridge) randomised controlled trial." BMJ: British Medical Journal 335 (7618):490. | UK | To provide insight into factors that contribute to the anxiety reported in a quantitative study of the psychological effect of screening for type 2 diabetes. To explore expectations of and reactions to the screening experience of patients with positive, negative, and intermediate results. | ADDITION (Cambridge) - a screening programme for type 2 diabetes in primary care. | Participants' perceptions changed as they progressed through the screening programme; the stepwise process seemed to help them adjust psychologically. The first screening test was typically considered unimportant and was attended with no thought about its implications. By the final diagnostic test, type 2 diabetes was considered a strong possibility, albeit a “mild” form. After diagnosis, people with screen detected type 2 diabetes tended to downplay its importance and talked confidently about their plans to control it. Participants with intermediate results seemed uncertain about their diagnosis, and those who screened negative were largely unaware of their remaining high risk. |
| Eborall, H., M. Stone, N. Aujla, N. Taub, M. Davies, and K. Khunti. 2012. "Influences on the uptake of diabetes screening: a qualitative study in primary care." Br J Gen Pract 62 (596):e204-11. doi: 10.3399/bjgp12X630106. | UK | To explore the perspectives of those invited to attend the MY-WAIST screening study for type 2 diabetes, particularly explanations for attending or not, and views on the specific screening strategy. | Primary-care-based screening programme for risk of type 2 diabetes followed by a screening appointment at the general practice, including an OGTT and waist measurement by a healthcare professional. | Two categories of influence on the decision about attending screening emerged. 1) Beliefs about type 2 diabetes candidacy and severity: perceived susceptibility to type 2 diabetes was more common amongs those who had attended; lack of perceived severity of type 2 diabetes was more common amongs those who did not attend. 2) Practical aspects about the screening strategy: the lengthy, early morning screening appointments were a barrier to uptake; screening attendees found the procedure largely acceptable. Prescreening waist self-measurement was more memorable than the remainder of the riskscore calculation; neither impacted on uptake |
| Ellis, N., C. Gidlow, L. Cowap, J. Randall, Z. Iqbal, and J. Kumar. 2015. "A qualitative investigation of non-response in NHS health checks." Arch Public Health 73 (1):14. doi: 10.1186/s13690-015-0064-1. | UK | to explore how non-attenders of NHS Health Checks perceive the programme, identify reasons for non-attendance and inform strategies to improve uptake | NHS Health Check: A 40-minute appointment, including anthropometrics, blood pressure, point-of-care cholesterol and glucose testing and lifestyle assessment, followed by tailored advice on lifestyle improvement. | Fundamental requirements for improving uptake are that individuals recognise the personal relevance of Health Checks and that attendance is convenient. Incorporating more sophisticated and personalised risk communication as part of the invitation could increase impact and promote candidacy. Flexibility and convenience of appointments should be considered by participating general practices. |
| Enocson, A., K. Jolly, R. E. Jordan, D. A. Fitzmaurice, S. M. Greenfield, and P. Adab. 2018. "Case-finding for COPD in primary care: A qualitative study of patients' perspectives." International Journal of COPD 13:1623-1632. doi: 103 http://dx.doi.org/10.2147/COPD.S147718. | UK | to explore patients' views and attitudes about COPD case-finding in general and on the processes involved | TargetCOPD trial, which compared active case-finding with routine care, in terms of yield (number of new cases of undiagnosed COPD detected).13 For the trial, eligible subjects were between 40 and 79 years, with a smoking history, and no prior diagnosis of COPD. Patients in the active case-finding arm received a screening questionnaire through their general practice, and those reporting relevant respiratory symptoms were then invited for diagnostic spirometry. Those with airflow obstruction on spirometry fulfilled the study criterion of COPD and had their results sent to their general practice. | The 43 interviews revealed the following two main categories of themes: patients’ views on COPD case-finding and barriers to case-finding. Overall, case-finding was deemed important and beneficial. Participants highlighted the need for screening activities to be convenient for patients but perceived that general practitioners (GPs) lacked the time and accessing appointments was difficult. Desire for a health check among symptomatic patients facilitated participation in case-finding. Psychological barriers to engagement included denial of ill health or failure to recognize symptoms, fear of the “test”, and lung symptoms being low on the hierarchy of patient health complaints. Mechanical barriers included providing care for another person (and therefore being too busy), being unable to access GP appointments, and lacking feedback of spirometry results or communication of the diagnosis. |
| Harkins, C., Shaw, R., Gillies, M., Sloan, H., Macintyre, K., Scoular, A., Morrison, C., MacKay, F., Cunningham, H., Docherty, P., Macintyre, P., Findlay, I. N. 2010. "Overcoming barriers to engaging socio-economically disadvantaged populations in CHD primary prevention: a qualitative study." BMC Public Health. | Scotland | To explore the barriers and facilitators to engaging a socioeconomically disadvantaged (SED) population in primary prevention for coronary heart disease (CHD). | Have a Heart Paisley (HaHP) offered risk screening to all eligible individuals. The programme employed two approaches to engaging with the community: a) a social marketing campaign and b) a community development project adopting primarily face-to-face canvassing. | Various reasons were identified for low uptake of risk screening amongst individuals living in areas of high SED in response to the social marketing campaign and a number of ways in which the face-to-face canvassing approach overcame these barriers were identified. These have been categorised into four main themes: (1) processes of engagement; (2) issues of understanding; (3) design of the screening service and (4) the priority accorded to screening. The most immediate barriers to recruitment were the invitation letter, which often failed to reach its target, and the general distrust of postal correspondence. In contrast, participants were positive about the face-to-face canvassing approach. Participants expressed a lack of knowledge and understanding about CHD and their risk of developing it and felt there was a lack of clarity in the information provided in the mailing in terms of the process and value of screening. In contrast, direct face-to-face contact meant that outreach workers could explain what to expect. Participants felt that the procedure for uptake of screening was demanding and inflexible, but that the drop-in sessions employed by the community development project had a major impact on recruitment and retention. |
| Jansen, Yvonne Jfm, Antoinette De Bont, Marleen Foets, Marc Bruijnzeels, and Roland Bal. 2007. "Tailoring intervention procedures to routine primary health care practice; an ethnographic process evaluation." BMC Health Services Research 7 (1):125. doi: 10.1186/1472-6963-7-125. | The Netherlands | To show how a prevention programme(Quattro project), which could be tailored to routine care, was implemented in primary care | Quattro project which is a prevention programme for cardiovascular diseases in high-risk patients in primary health care centres in deprived neighbourhoods in Rotterdam and the Hague. The core of the Quattro project was the collaboration between a practice nurse, a peer health educator, the GP, and assistant (hence Quattro care) in providing intensified preventive care. Multidisciplinary patient care teams are thought to improve the quality of care in general practice, as they are seen as a means of relieving the workload of GPs and to assist them in providing preventive activities | The ethnographic process evaluation showed that the opportunity of tailoring intervention procedures to routine care in a pragmatic trial setting did not result in a wellorganised and well-implemented prevention programme. In fact, the lack of standard protocols hindered the implementation of the intervention. Although it was not the purpose of this trial, a guideline was developed. Despite the fact that the developed guideline functioned as a tool, it did not result in the intervention being organised accordingly. However, the guideline did make tailoring the intervention possible. It provided the professionals with the key or the instructions needed to achieve organisational change and transform the existing interprofessional relations. |
| Jansen, Yvonne Jfm, Roland Bal, Marc Bruijnzeels, Marleen Foets, Rianne Frenken, and Antoinette De Bont. 2006. "Coping with methodological dilemmas; about establishing the effectiveness of interventions in routine medical practice." BMC Health Services Research 6 (1). doi: 10.1186/1472-6963-6-160. | The Netherlands | The aim of this paper is to show how researchers balance between scientific rigour and localisation in conducting pragmatic trial research. | Quattro project which is a prevention programme for cardiovascular diseases in high-risk patients in primary health care centres in deprived neighbourhoods in Rotterdam and the Hague. The core of the Quattro project was the collaboration between a practice nurse, a peer health educator, the GP, and assistant (hence Quattro care) in providing intensified preventive care. Multidisciplinary patient care teams are thought to improve the quality of care in general practice, as they are seen as a means of relieving the workload of GPs and to assist them in providing preventive activities | Conducting a pragmatic trial is a continuous balancing act between meeting methodological demands and implementing a complex intervention in routine primary health care. As an effect, the research design had to be adjusted pragmatically several times and the intervention that was meant to be tailor-made became a rather stringent procedure. |
| Liljas, A. E. M., K. Walters, A. Jovicic, S. Iliffe, J. Manthorpe, C. Goodman, and K. Kharicha. 2019. "Engaging 'hard to reach' groups in health promotion: the views of older people and professionals from a qualitative study in England." BMC Public Health 19 (1):629. doi: 10.1186/s12889-019-6911-1. | UK | This study aimed to explore what influences them practicing health promotion and elicit the views of cross-sector professionals with experiences of working with ‘hard to reach’ older people, to help inform best practice on engagement. | N/R | Older people’s motivation to stay healthy and independent reflected their everyday behaviour including practicing activities to feel or stay well, level of social engagement, and enthusiasm for and belief in health promotion. All of the oldest old reported trying to live healthily, often facilitated by others, yet sometimes being restricted due to poor health. Most older people from BME groups reported a strong wish to remain independent which was often positively influenced by their social network. Older people living in deprived areas reported reluctance to undertake health promotion activities, conveyed apathy and reported little social interaction. Cross-sector health professionals consistently reported similar themes as the older people, reinforcing the views of the older people through examples. |
| Olaya-Contreras, P., K. Balcker-Lundgren, F. Siddiqui, and L. Bennet. 2019. "Perceptions, experiences and barriers to lifestyle modifications in first-generation Middle Eastern immigrants to Sweden: a qualitative study." BMJ Open 9 (10):e028076. doi: 10.1136/bmjopen2018-028076. | Sweden | The aim of the present study was to explore perceptions, experiences and barriers concerning lifestyle modifications (LSM) in Iraqi immigrants to Sweden at risk for T2D. | a culturally adapted intervention, the MEDIM, impact of Migration and Ethnicity on Diabetes in Malmö cohort study 2010–2012. The intervention group were offered to participate in a total of seven gender-specific group sessions, which were conducted in their native language. The sessions addressed awareness, motivation, taking action, relapse, review and feedback, setting goals and self-empowerment. | Participants expressed awareness of the content of healthy lifestyle practices. They also expressed numerous social and cultural barriers to LSM connected to irregular meals, overeating, food and drinking preferences and family expectations. Overeating was described as a consequence of social and cultural norms and expectations and of poor mental well-being. Facilitators for reaching successful LSM were connected to family involvement and support |
| Riley, R., N. Coghill, A. Montgomery, G. Feder, and J. Horwood. 2015. "The provision of NHS health checks in a community setting: an ethnographic account." BMC Health Serv Res 15:546. doi: 10.1186/s12913-015-1209-1. | UK | This study aimed to examine the feasibility and acceptability of community outreach NHS Health Checks targeted at the Afro-Caribbean community | NHS Health Check: A 40-minute appointment, including anthropometrics, blood pressure, point-of-care cholesterol and glucose testing and lifestyle assessment, followed by tailored advice on lifestyle improvement. | Analysis revealed the value of community assets (community engagement workers, churches, and community centres) to publicise the event and engage community members. People were motivated to attend for preventative reasons, often prompted by familial experience of cardiovascular disease. Attendees valued outreach NHS Health Checks, reinforcing or prompting some to make healthy lifestyle changes. The NHS Health Check provided an opportunity for attendees to raise other health concerns with health staff and to discuss their test results with peers. For some participants, the communication of test results, risk and lifestyle information was confusing and unwelcome. The findings additionally highlight the need to ensure community venues are fit for purpose in terms of assuring confidentiality. |
| Shaw, R. L., H. Lowe, C. Holland, H. Pattison, and R. Cooke. 2016. "GPs' perspectives on managing the NHS Health Check in primary care: a qualitative evaluation of implementation in one area of England." BMJ Open 6 (7):e010951. doi: 10.1136/bmjopen-2015-010951. | UK | To evaluate the implementation of the National Health Service (NHS) Health Check programme in one area of England from the perspective of general practitioners (GPs). | NHS Health Check: A 40-minute appointment, including anthropometrics, blood pressure, point-of-care cholesterol and glucose testing and lifestyle assessment, followed by tailored advice on lifestyle improvement. | Themes were generated which represent GPs' experiences of managing the NHS Health Check: primary care as a commercial enterprise; ‘buy in’ to concordance in preventive healthcare; following protocol and support provision. These themes represent the key issues raised by GPs. They reveal variability in the implementation of NHS Health Checks. GPs also need support in allocating resources to the Health Check including training on how to conduct checks in a concordant (or collaborative) way. |
| Stol, Yrrah H., Eva C. Asscher, and Maartje H. Schermer. 2018. "Good health checks according to the general public; expectations and criteria: A focus group study." BMC Medical Ethics Vol 19 2018, ArtID 64 19. | The Netherlands | To explore what participants consider characteristics of good and bad health checks, and whether they saw a role for the Dutch government. | N/R | Participants consider a good predictive value the most important characteristic of a good health check. Information before, during and after the test, knowledgeable and reliable providers, tests for treatable (risk factors for) disease, respect for privacy, no unnecessary health risks and accessibility are also mentioned as criteria for good health checks. Participants make many assumptions about health check offers. They assume health checks provide certainty about the presence or absence of disease, that health checks offer opportunities for health benefits and that the privacy of health check data is guaranteed. In their choice for provider and test they tend to rely more on heuristics than information. Participants trust physicians to put the interest of potential health check users first and expect the Dutch government to intervene if providers other than physicians failed to do so by offering tests with a low predictive value, or tests that may harm people, or by infringing the privacy of users. |
| Stuber, J. M., C. N. H. Middel, J. D. Mackenbach, J. W. J. Beulens, and J. Lakerveld. 2020. "Successfully Recruiting Adults with a Low Socioeconomic Position into Community-Based Lifestyle Programs: A Qualitative Study on Expert Opinions." Int J Environ Res Public Health 17 (8). doi: 10.3390/ijerph17082764. | The Netherlands | To explore experts’ perceived challenges and success factors in the recruitment of adults with a low socioeconomic position (SEP) for participation in community-based lifestyle modification programs | No intervention | Results revealed challenges related to the context of the program (e.g., limited program resources), psychosocial barriers of the participants (e.g., mistrust or skepticism), practical barriers (e.g., low literacy or having other priorities), and reasons to decline participation (e.g., lack of interest or motivation). Success factors were related to securing beneficial contextual and program-related factors (e.g., multi-layered recruitment strategy), establishing contact with the target group (e.g., via existing networks, community key-members), methods to increase engagement (e.g., personal approach and involvement of the target group in the program process) and making participation easier (e.g., providing transport), and providing various types of incentives. |
| Tonnon, S. C., Proper, K. I., van der Ploeg, H. P., Westerman, M. J., Sijbesma, E., van der Beek, A. J. 2014. "A qualitative study of the anticipated barriers and facilitators to the implementation of a lifestyle intervention in the dutch construction industry." BMC Public Health 14. doi: 10.1186/1471-2458-14-1317 | The Netherlands | This qualitative study sought to determine anticipated barriers and facilitators to the nationwide implementation of an effective lifestyle intervention in the construction industry in the Netherlands. | N/R | Hypothetical employee willingness to sign up for the intervention was facilitated by a high level of perceived risk, perceived added value of the intervention, and perceived social support. It was hampered by a preference for independence and perceived interference with their work. All professionals named a lack of time as an anticipated barrier to implementation. Lifestyle counselors suggested several strategies to improve the proficiency of their counseling technique, such as training in small groups and a continuous stream of employee referrals. Occupational physicians thought they would be hampered in screening employees and referring them to a lifestyle counselor by the perception that addressing employee lifestyles was not their task, and by a counter-productive relationship with other stakeholders. The manager addressed financial incentives and a good intervention fit with the current approach of the OHS. |
| Usher-Smith, J. A., L. R. Winther, G. S. Shefer, B. Silarova, R. A. Payne, and S. J. Griffin. 2017. "Factors Associated With Engagement With a Web-Based Lifestyle Intervention Following Provision of Coronary Heart Disease Risk: Mixed Methods Study." J Med Internet Res 19 (10):e351. doi: 10.2196/jmir.7697 | UK | The study aimed to explore individual-level factors associated with different degrees of engagement with a Web-based behavior change intervention following provision of coronary heart disease (CHD) risk information, and the barriers and facilitators to engagement. | INFORM was a parallel group randomized controlled trial that aimed to explore the short-term effects on health-related behaviors of giving people different types of information online about their estimated risk of CHD in the subsequent 10 years, together with Web-based lifestyle advice | Low engagement was more often associated with: (1) reporting a negative emotional reaction in response to the risk score (P=.029), (2) perceiving that the intervention did not provide any new lifestyle information (P=.011), and (3) being less likely to have reported feeling an obligation to complete the intervention as part of the study (P=.019). The mixed-methods matrix suggested that there was also an association between low engagement and less success with previous behavior change attempts, but the statistical evidence for this association was weak (P=.16). No associations were seen between engagement and barriers or facilitators to health behavior change, or comments about the design of the intervention itself. The most commonly cited barriers related to issues with access to the intervention itself: either difficulties remembering the link to the site or passwords, a perceived lack of flexibility within the website, or lack of time. Facilitators included the nonjudgmental presentation of lifestyle information, the use of simple language, and the personalized nature of the intervention. |
| Voogdt-Pruis, H. R., G. H. Beusmans, A. P. Gorgels, and J. W. van Ree. 2011. "Experiences of doctors and nurses implementing nurse-delivered cardiovascular prevention in primary care: a qualitative study." J Adv Nurs 67 (8):1758-66. doi: 10.1111/j.1365- 2648.2011.05627.x. | The Netherlands | To explore the experiences of general practitioners and practice nurses implementing nurse-delivered cardiovascular prevention to high risk patients in primary care. | Nurse-delivered cardiovascular prevention to high risk patients in primary care | Main barriers to the implementation included: lack of knowledge about the guideline, attitudes towards treatment targets, lack of communication, insufficient coaching by doctors, content of life style advice. At the start of the consultation project, practice nurses expressed concern of losing nursing tasks. Other barriers were related to patients (lack of motivation), the guideline (target population) and organizational issues (insufficient patient recording and computer systems). |
